# CXCR7 activation evokes the anti-PD-L1 antibody against glioblastoma by remodeling CXCL12-mediated immunity

**DOI:** 10.1038/s41419-024-06784-6

**Published:** 2024-06-19

**Authors:** Chan-Chuan Liu, Wen-Bin Yang, Chia-Hung Chien, Cheng-Lin Wu, Jian-Ying Chuang, Pin-Yuan Chen, Jui-Mei Chu, Siao Muk Cheng, Li-Ying Qiu, Yung-Chieh Chang, Daw-Yang Hwang, Chih-Yuan Huang, Jung-Shun Lee, Kwang-Yu Chang

**Affiliations:** 1 National Institute of Cancer Research, National Health Research Institutes, Tainan, Taiwan; 2 Research Center for Neuroscience, Taipei Medical University, Taipei, Taiwan; 3 Ph.D. Program in Medical Neuroscience, College of Medical Science and Technology, Taipei Medical University, Taipei, Taiwan; 4 School of Medicine, I-Shou University, Kaohsiung, Taiwan; 5 Department of Pathology, National Cheng Kung University Hospital, College of Medicine, National Cheng-Kung University, Tainan, Taiwan; 6 International Master Program in Medical Neuroscience, Taipei Medical University, Taipei, Taiwan; 7 Department of Biomedical Science and Environmental Biology, Kaohsiung Medical University, Kaohsiung, Taiwan; 8 School of Medicine, Chang Gung University, Taoyuan, Taiwan; 9 Department of Neurosurgery, Keelung Chang Gung Memorial Hospital, Keelung, Taiwan; 10 TMU Research Center of Cancer Translational Medicine; Taipei Cancer Center; Taipei Medical University Hospital, College of Medicine, Taipei Medical University, Taipei, Taiwan; 11 Division of Neurosurgery, Department of Surgery, National Cheng Kung University Hospital, College of Medicine, National Cheng Kung University, Tainan, Taiwan; 12 Department of Oncology, National Cheng Kung University Hospital, College of Medicine, National Cheng Kung University, Tainan, Taiwan; 13 Center of Cell Therapy, National Cheng Kung University Hospital, College of Medicine, National Cheng Kung University, Tainan, Taiwan

**Keywords:** CNS cancer, Tumour immunology, Cancer microenvironment

## Abstract

The interaction between glioblastoma cells and glioblastoma-associated macrophages (GAMs) influences the immunosuppressive tumor microenvironment, leading to ineffective immunotherapies. We hypothesized that disrupting the communication between tumors and macrophages would enhance the efficacy of immunotherapies. Transcriptomic analysis of recurrent glioblastoma specimens indicated an enhanced neuroinflammatory pathway, with CXCL12 emerging as the top-ranked gene in secretory molecules. Single-cell transcriptome profiling of naïve glioblastoma specimens revealed CXCL12 expression in tumor and myeloid clusters. An analysis of public glioblastoma datasets has confirmed the association of CXCL12 with disease and PD-L1 expression. In vitro studies have demonstrated that exogenous CXCL12 induces pro-tumorigenic characteristics in macrophage-like cells and upregulated PD-L1 expression through NF-κB signaling. We identified CXCR7, an atypical receptor for CXCL12 predominantly present in tumor cells, as a negative regulator of CXCL12 expression by interfering with extracellular signal-regulated kinase activation. CXCR7 knockdown in a glioblastoma mouse model resulted in worse survival outcomes, increased PD-L1 expression in GAMs, and reduced CD8^+^ T-cell infiltration compared with the control group. Ex vivo T-cell experiments demonstrated enhanced cytotoxicity against tumor cells with a selective CXCR7 agonist, VUF11207, reversing GAM-induced immunosuppression in a glioblastoma cell-macrophage-T-cell co-culture system. Notably, VUF11207 prolonged survival and potentiated the anti-tumor effect of the anti-PD-L1 antibody in glioblastoma-bearing mice. This effect was mitigated by an anti-CD8β antibody, indicating the synergistic effect of VUF11207. In conclusion, CXCL12 conferred immunosuppression mediated by pro-tumorigenic and PD-L1-expressing GAMs in glioblastoma. Targeted activation of glioblastoma-derived CXCR7 inhibits CXCL12, thereby eliciting anti-tumor immunity and enhancing the efficacy of anti-PD-L1 antibodies.

## Introduction

Glioblastoma is the most prevalent and life-threatening primary brain tumor and is characterized by histopathological features [1]. Recent advances in genetic signature analysis have contributed to precise classification based on the WHO criteria and have also revealed molecular subtypes according to tumor heterogeneity [1]. The standard treatment involves neurosurgical removal, followed by temozolomide-based concurrent chemoradiotherapy. Despite initial treatment advances leading to enhanced survival and the introduction of innovative anti-cancer drugs leading to little success, permanent effects remain limited. Consequently, the median survival time is only 15-18 months following treatments [1].

Immune checkpoint blockades (ICBs) are groundbreaking anti-cancer drugs designed to re-evoke the anti-tumor immune responses by blocking immune checkpoints (ICs), such as PD-1/PD-L1 [2]. In certain tumors, ICBs represent a breakthrough owing to its distinct mechanisms and potential long-term benefits. However, in other malignancies, including in glioblastoma, various factors can counteract ICB effects, leading to treatment failure [3–6]. Glioblastoma-associated macrophages (GAMs), also known as tumor-associated macrophages in glioblastoma, play a crucial role in shaping the tumor microenvironment (TME) which is composed of extracellular matrix, resident stromal cells, and recruited cells. GAMs constitute 30% of the glioblastoma mass, and tumor-secreting factors can mediate their activation into different functional classes [7, 8]. GAMs have been implicated in resistance to anti-tumor therapies and disease progression [9, 10]. Notably, reprogramming of tumor-associated macrophages by interacting with TME can enhance their pro-tumorigenic features such as creating a chronic inflammatory environment, inducing mesenchymal transition in resident cells, and supporting tumor-associated angiogenesis. It can also remodel the extracellular matrix to form barriers against lymphocyte infiltration [11]. Altogether, the factor is crucial in causing the immunosuppression within tumor diseases.

CXCL12, also known as stromal-derived factor-1, is a chemoattractant and chemokine that regulates various physiological processes. With its conventional receptor, CXCR4, CXCL12 modifies immunity by immune cell recruitment and polarization, such as altering tumor-associated macrophages and T cells, eventually promoting malignancies [12–15]. Glioblastoma-derived CXCL12 forms an autocrine-positive feedback loop through the tumor-bound CXCR4 in response to environmental stress [16]. Interestingly, CXCR7 serves as an atypical G-protein-coupled receptor for CXCL12, counterbalancing CXCL12 in glial cells and protecting against neuroinflammatory diseases in nervous system [17]. In glioblastoma, it was reported for the upregulated CXCR7 expression in tumor cells and tumor-associated vasculature during disease progression [18]. Although the exact role of CXCR7 remains uncertain, it is generally known to be involved in the regulation and function of CXCL12.

Considering that glioblastoma cells have the potential to shape the immunosuppressive TME through interactions with GAM, we hypothesized that disrupting the tumor-to-macrophages communication could enhance the effectiveness of ICB therapy by triggering anti-tumor immunity. By integrating the transcriptome data from clinical specimens, in vitro, and in vivo approaches, we unveiled the importance and therapeutic potential of the regulatory mechanisms of CXCR7 on CXCL12 in glioblastoma cells, which consequently affected GAM induction and T-cell alteration.

## Materials and methods

### Analysis of RNA sequencing data

Bulk transcriptomic data from 14 paired naïve-recurrent glioma specimens were obtained from our previous study [19]. The re-utilization of these data was approved by the Institutional Review Board of the National Health Research Institute (NHRI-EC1100206). Differential expression analysis, pathway analysis, and the significant change of gene expression were performed as previously described [20].

### Analysis of single-cell RNA sequencing data

The preparation of single cells and RNA-barcode library from clinical specimens were detailed in previous study [19]. The data analysis were performed using the Loupe Browser 6.0 (http://software.10xgenomics.com/single-cell/overview/welcome) with the criteria set for the exclusion of the unexpected unique molecule index (UMI; UMI: <200 or >20,000), feature expression (genes: <100 or >4000), and poor quality or dying cells (mitochondrial genes: >15%).

### Analysis of online clinical datasets

We examined CXCL12 expression in The Cancer Genome Atlas (TCGA) glioblastoma dataset using UALCAN (http://ualcan.path.uab.edu/analysis.html) and the Chinese Glioma Genome Atlas (CGGA; http://www.cgga.org.cn/index.jsp). The correlation between CXCL12 and PD-L1 in the CGGA dataset was analyzed using GlioVis (http://gliovis.bioinfo.cnio.es/).

### Cell culture

Human glioblastoma cell lines U87MG and A172, human leukemic cell line THP-1, mouse glioblastoma cell line GL261, and mouse leukemia cell line Raw264.7 were purchased from the American Type Culture Collection (VA, USA). U87MG, A172, GL261, Raw264.7, and glioblastoma patient-derived Pt#3 cells from a wild-type-IDH1 glioblastoma that was originated in a 32-year-old female patient [21], were maintained in DMEM high-glucose medium (GeneDirex, Taiwan) with 100 unit/mL penicillin, 100 unit/mL streptomycin (P/S; Gibco, CA, USA), and 10% heat-inactivated fetal bovine serum (hiFBS; Hyclone, UT, USA). THP-1 cells were maintained in RPMI1640 (Gibco) with P/S and 10% hiFBS. The cells were incubated at 37 °C in a humidified 95% air/5% CO_2_.

### qRT-PCR

Total RNA was isolated using TRIzol (Invitrogen, CA, USA) or an RNA isolation kit (GeneDirex) following a standard procedure and subjected to PCR using SuperScript II reagent (Invitrogen). Complementary DNA was mixed with the SYBR Green Master Mix (Applied Biosystems, CA, USA) and specific primers (Supplementary Table S1). Data were normalized to GAPDH using the 2^-ΔΔCT^ formula.

### RNA-based gene modulation

Lipofectamine RNAiMAX reagent (Invitrogen) was used for the transfection of siRNA (Supplementary Table S2) following the manufacturer’s instructions.

### Cytotoxicity MTT assay

The cells were incubated with 5% 3-(4,5-dimethylthiazol-2-yl)-2,5-diphenyltetrazolium bromide (MTT; Merck, NJ, USA) for 2 h. DMSO was used to solubilize the purple formazan crystals, and the absorbance at 570 nm was measured.

### Macrophage differentiation

THP-1 and Raw264.7 cells were differentiated into macrophage-like cells (MLCs) by incubating with 200 nM phorbol-12-myristate-13-acetate (PMA; Merck) for 72 h.

### WST cell proliferation assay

The WST solution (Takara Bio, Kusatsu, Japan) was added to the treated cells. The absorbance at 440 nm was measured.

### Flow cytometry

The cells labeled with multiple fluorochrome-conjugated antibodies (Supplementary Table S3) were detected using Attune NxT flow cytometry (Thermo-Fisher Scientific, MA, USA). Attune NxT Software (Thermo-Fisher Scientific) and GraphPad Prism 6.01 software (Prism) were used for data analysis.

### Orthotopic mouse glioblastoma model

Animal experiments were approved by the Institutional Animal Care and Use Committee of the National Health Research Institute (NHRI-IACUC-109187 and 112066). For tumorigenesis, 50,000 GL261 cells carrying shRNA or parental GL261 cells were injected intracranially into the striatum of 8–12-week-old female C57BL/6NCrlBltw mice (*N* = 10 for each group; BioLASCO, Taiwan) [22]. Twice-weekly intraperitoneal administration of VUF (MedChemExpress, CA, USA) [23], DMSO, and antibodies (BioXcell, NH, USA) (Supplementary Table S2) [24] was initiated on post-implantation day 7. The survival and body weight were analyzed using Prism. When mice were sacrificed, a part of the tumor-bearing brains was dissociated into single-cell suspensions using the Brain Tumor Dissociation Kit (Miltenyi Biotec, Cologne, Germany), followed by Myelin Removal (Miltenyi Biotec) for flow cytometry analysis, whereas the remaining part was fixed in paraformaldehyde and embedded in paraffin for staining.

### Hematoxylin-eosin staining and immunohistochemistry staining

Paraffin-embedded sections were stained with hematoxylin-eosin (H&E) and immunohistochemistry (IHC) as previously described [22]. IHC staining was performed using the ImageJ software (http://rsbweb.nih.gov/ij/). Specific cells were identified by a senior pathologist.

### Ex vivo T cell cytotoxic assay

GL261 cells stably expressing luciferase/turboGFP (GL261-Luc/tGFP) were developed as previously described [22]. GAMs were induced by co-culturing the mouse MLCs with GL261-Luc/tGFP cells at a ratio of 1:4 for 1 day. Pan T cells (PTCs) were collected from the spleens of the intracranially GL261-bearing mice and isolated using PTC Isolation Kit (Miltenyi Biotec). PTCs were rested for 1 day in RPMI-1640 medium (Gibco) with 100 unit/ml mouse IL-2 (PeproTech, NJ, USA), 50 μM β-mercaptoethanol (Gibco), P/S (Gibco), and 10% hiFBS (Hyclone). A co-culture with a ratio of 1:4:20 of GAMs, GL261-Luc/tGFP cells, and PTCs was treated for 48 h. The suspended cells were collected for flow cytometry. The luminescence of adhered GL261-Luc/tGFP cells was measured using the ONE-Glo™ Luciferase Assay System (Promega, WI, USA). T-cell cytotoxicity was calculated using the following formula: % = [(luminescence of GL261-Luc/tGFP cells in control) − (luminescence of GL261-Luc/tGFP cells with PTCs and GAMs in control or treatment) × 100]/(luminescence of GL261-Luc/tGFP cells in control) [25].

### Statistical analysis

Data are presented as mean ± standard error of the mean and statistically analyzed using Prism or Excel 2013 (Microsoft). The differences between two variables were calculated using an unpaired two-tailed *t* test or one-way analysis of variance (ANOVA), as appropriate. Survival was compared between the groups using the log-rank (Mantel–Cox) test. *p* < 0.05 was considered significant.

## Results

### CXCL12 was positively correlated with PD-L1 and glioblastoma progression

**Fig. 1:**
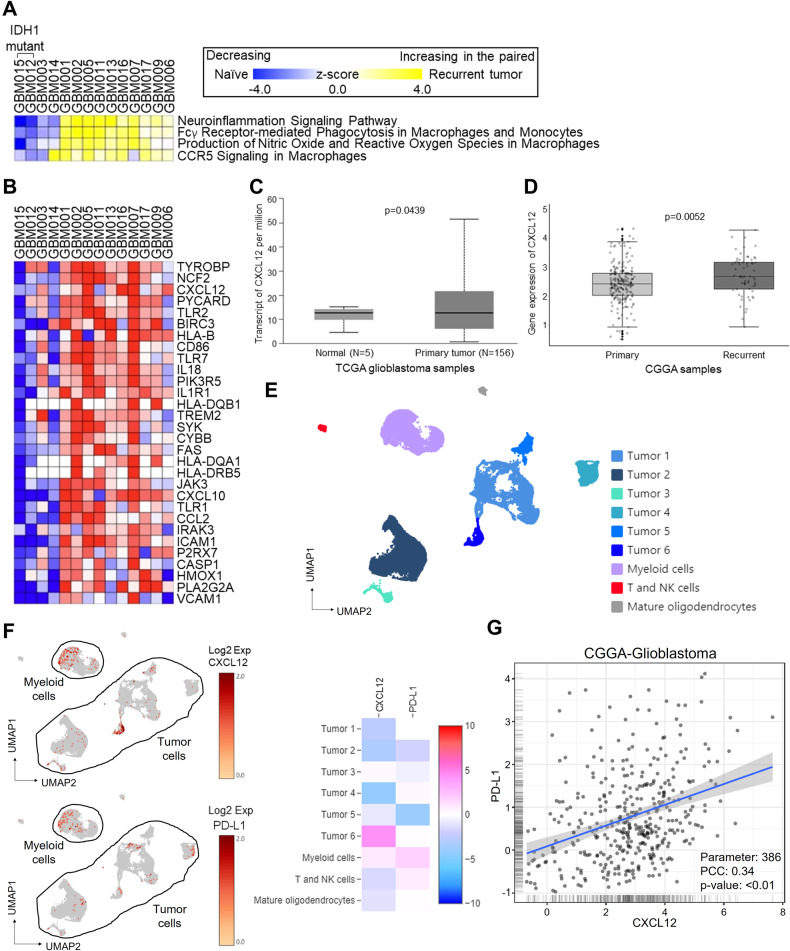
Glioblastoma progression was accompanied by increased CXCL12 in glioblastoma cells and myeloid cells. **A** Pathway analysis of RNA array of 14 paired naïve-recurrent specimens from high-grade glioma patients, including 2 pairs with grade III features (GBM 001 and 002) and 2 pairs with IDH1 mutation (GBM 012 and 015); **B** The fold change of individual genes of the neuroinflammation signaling pathway by a heatmap in the recurrent gliomas; **C** CXCL12 expression in TCGA-glioblastoma dataset; **D** CXCL12 expression in CGGA-mRNA325 dataset; **E** UMAP of cell annotation of naïve glioblastoma patient-derived scRNA seq dataset; **F** the expression distribution and heatmap of CXCL12 and PD-L1 in naïve glioblastoma patient-derived scRNA seq dataset; **G** The correlation of CXCL12 and PD-L1 in CGGA dataset. CXCL12 expression in **B** and **C** was compared by using an unpaired *t* test. The correlation of CXCL12 and PD-L1/CD274 was analyzed by the Pearson correlation coefficient.

To identify the critical factor in the interaction between tumor cells and GAMs, we first examined the bulk transcriptomic profiles of our clinically paired naïve-recurrent glioma specimens. Ingenuity pathway analysis revealed an enhanced neuroinflammatory pathway in recurrent grade IV tumors compared with their treatment-naïve counterparts, with no such distinction found for grade III gliomas (Fig. 1A). Further examination of individual genes indicated CXCL12 as a top-ranking increased secretion factor in the neuroinflammatory pathway (Fig. 1B). The CXCL12 expression in datasets from TCGA-glioblastoma and CGGA-mRNA-325 indicated elevated levels of CXCL12 in glioblastoma compared with normal tissues (Fig. 1C) and in recurrent gliomas compared with treatment-naïve gliomas (Fig. 1D). Subsequently, to explore the regulatory role of CXCL12 in GAMs, we analyzed the expression of CXCL12 and IC PD-L1 in a single-cell RNA sequencing (scRNA-seq) dataset obtained from treatment-naïve glioblastoma specimens. The UMAP and heatmap indicated that CXCL12 was predominantly expressed in specific tumor clusters, including Tumor 3 and Tumor 6, as well as in myeloid-cell clusters, while PD-L1 was predominantly expressed in myeloid-cell cluster (Fig. 1E, F). Datasets from CGGA and our paired grade IV gliomas consistently revealed that CXCL12 was positively correlated with PD-L1 expression (Fig. 1G and Supplementary Fig. S1B).

### CXCL12 induced PD-L1 expression in macrophage-like cells through the NF-κB pathway

To investigate the effect of CXCL12 on macrophage characteristics, three MLCs (THP-1, SC, and Raw264.7) and a human microglial cell line (HMC-3) were used, and exogenous CXCL12 were applied to these cells. Notably, a high concentration of CXCL12 (100 ng/mL; CXCL12^hi^), but not a low concentration (10 ng/mL; CXCL12^low^), promoted the MLC proliferation (Supplementary Fig. S2A). Subsequently, in the human THP-1 model, CXCL12^hi^ increased interleukin (IL)-6 and IL1R1 expression (Supplementary Fig. S2B). In contrast, CXCL12^low^ reduced IL-1β, MMP9, MRC1, and IL1R2 expression, whereas CXCL12^hi^ did not affect these genes (Supplementary Fig. S2B). Similarly, in a mouse Raw264.7 model, CXCL12^hi^ upregulated IL-6, IL1R1, MMP9, and IL1R2 expression (Supplementary Fig. S2C). These findings suggested that excess CXCL12 transforms macrophages into the pro-tumorigenic phenotype.

**Fig. 2:**
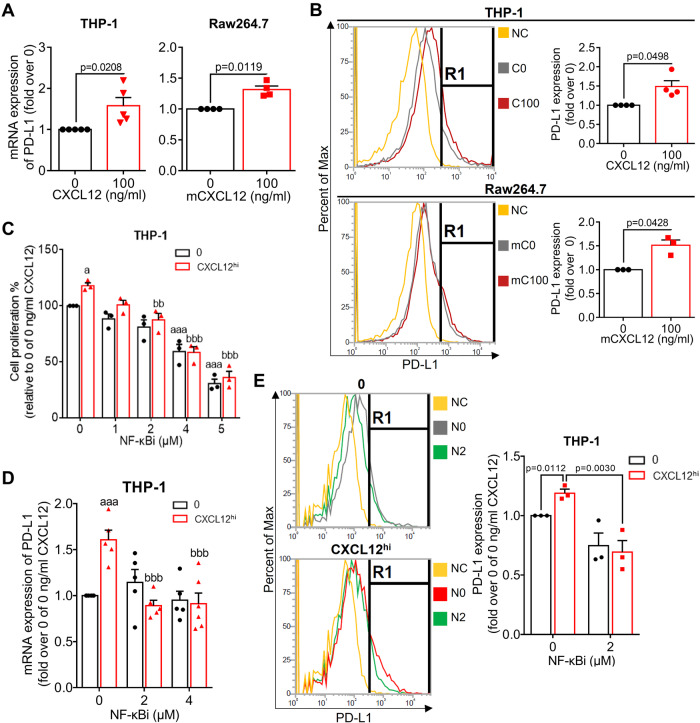
Glioblastoma-derived CXCR7-CXCL12 axis induced PD-L1 expression in GAMs through NF-κB signaling. **A** mRNA expression of PD-L1 in differentiated human THP-1 cells and mouse Raw264.7 cells in the presence of exogenous CXCL12; **B** flow cytometry indicating the cell membrane expression of PD-L1 in differentiated THP-1 cells and Raw264.7 cells in the presence of exogenous CXCL12; **C** The proliferation with NF-κBi BAY11-7082 in the absence or the presence of CXCL12; **D** mRNA expression of PD-L1 with NF-κBi in the absence or the presence of CXCL12^hi^; **E** Cell membrane expression of PD-L1 with 2 μM NF-κBi in the absence or the presence of CXCL12^hi^. PD-L1 expression in Fig. 2A, B was compared between in the absence and the presence of CXCL12^hi^ by using an unpaired *t* test. Statistical differences in Fig. 2C–E were assessed using one-way ANOVA. a Indicated the comparison with 0 ng/mL CXCL12. b indicated the comparison with 0 μM NF-κBi. a, b, *p* < 0.05; bb, *p* < 0.01; aaa, bbb, *p* < 0.001.

Next, we investigated whether excess CXCL12 influenced PD-L1 expression. Exogenous CXCL12^hi^ increased PD-L1 at mRNA and membrane protein levels in both MLCs and microglial models (Fig. 2A, B and Supplementary Fig. S2D). NF-κB has been reported to be involved in the CXCL12 signaling cascade and regulation of PD-L1 expression [26, 27]. Therefore, the selective NF-κB inhibitor BAY117082 (NF-κBi) was used. CXCL12^hi^ activated NF-κB signaling by reducing IκBα and increasing the phosphorylation on p65 while additional NF-κBi reversed CXCL12^hi^-activated NF-κB signaling (Supplementary Fig. S2E). We found that NF-κB inhibition reversed CXCL12^hi^-induced MLC proliferation (Fig. 2C) and upregulation of PD-L1 at both mRNA and membrane protein levels (Fig. 2D, E). Notably, in the absence of exogenous CXCL12, incubation with NF-κBi alone did not affect MLC proliferation or PD-L1 expression, indicating the pivotal role of CXCL12 in the CXCL12-NF-κB-PD-L1 axis.

### CXCR7 negatively regulated CXCL12 expression in glioblastoma cells and CXCL12-mediated PD-L1 expression in macrophages

**Fig. 3:**
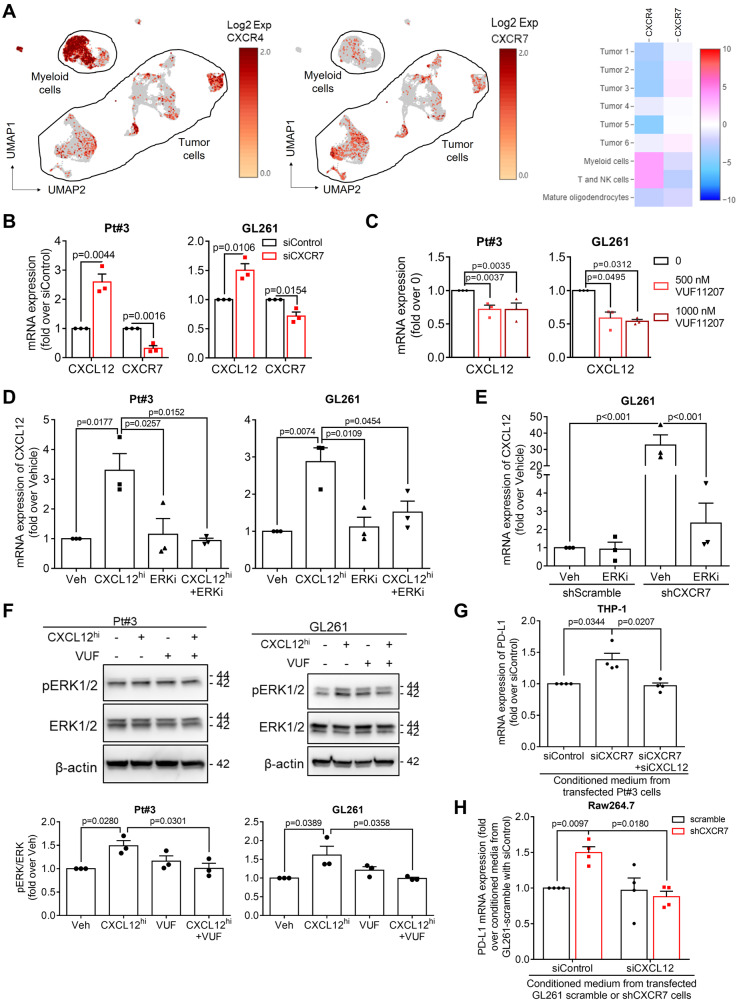
CXCR7 negatively regulated CXCL12 expression in glioblastoma cells. **A** The expression distribution and heatmap of CXCR4 and CXCR7 in naïve glioblastoma patient-derived scRNA seq dataset; **B** mRNA expression of CXCL12 and CXCR7 in the presence of siRNA of CXCR7; **C** mRNA expression of CXCL12 in the presence of CXCR7 agonist, VUF11207 (VUF); **D** mRNA expression of CXCL12 in the presence of CXCL12^hi^ and/or 5 μM ERKi U0126 in parental glioblastoma cells; **E** mRNA expression of CXCL12 in the presence of 5 μM ERKi in GL261 carrying shRNA of scramble or CXCR7; **F** Protein expression level of phosphorylated and total ERK in the presence of CXCL12^hi^ and/or 500 nM VUF; **G** mRNA expression of PD-L1 in differentiated THP-1 cells with the conditioned medium of CXCR7 knockdown or combining with CXCL12 knockdown by siRNA in parental Pt#3 cells; **H** mRNA expression of PD-L1 in MLCs cells with the conditioned medium of CXCL12 knockdown by siRNA in GL261 cells carrying shRNA of scramble or CXCR7. mRNA and protein expression of CXCL12 and CXCR7 was compared between siControl and siCXCR7 cells in **B** by using an unpaired *t* test. Statistical differences in **C**–**H** were assessed using one-way ANOVA.

We examined the CXCL12 receptors, CXCR4 and CXCR7. In the UMAP and heatmap of the scRNA-seq dataset, CXCR7 was predominantly expressed in tumor-cell clusters, particularly in Tumors 2, 3, and 6, whereas CXCR4 was predominantly expressed in both myeloid-cell and T-cell clusters (Fig. 3A). Consistently, upregulation of CXCL12 and downregulation of CXCR7 were observed in temozolomide-resistant glioblastoma U87MG, A172, and Pt#3 cells, without consistent change in CXCR4 expression (Supplementary Fig. S3A). Western blot analysis further revealed lower CXCR7 expression and higher CXCR4 expression in U87MG-R cells than in the parental counterpart (Supplementary Fig. S3B). These data suggested that CXCL12 and CXCR7 are associated with disease progression. To explore the regulatory role of CXCR7 in CXCL12, we suppressed CXCR7 expression in the parental cells using siRNA. The results consistently revealed enhanced CXCL12 expression, but not CXCR4 (Fig. 3B and Supplementary Fig. S3C). To further investigate the impacts of CXCR7, we established GL261 cells stably expressing shCXCR7 (GL261-shCXCR7). Consistently, GL261-shCXCR7 cells exhibited higher CXCL12 expression (Supplementary Fig. S3D). Conversely, CXCR7 overexpression in temozolomide-resistant U87MG-R and Pt#3-R cells decreased CXCL12 expression (Supplementary Fig. S3E). In human glioblastoma U87MG, Pt#3 cells, and mouse glioblastoma GL261 cells, CXCR7 agonist VUF11207 (VUF) similarly led to a reduction in CXCL12 expression (Fig. 3C and Supplementary Fig. S3F). Subsequently, exogenous CXCL12^hi^ enhanced the CXCL12 expression, which was mitigated by U0126, an extracellular-signal-regulated kinase (ERK) inhibitor (ERKi; Fig. 3D and Supplementary Fig. S3G) [28]. CXCR7 knockdown-induced CXCL12 expression was inhibited by ERKi (Fig. 3E). Notably, VUF partially reversed CXCL12-induced phosphorylation of ERK, but VUF alone did not show this effect (Fig. 3F and Supplementary Fig. S3H). These findings suggested that CXCR7 negatively regulates CXCL12 expression in glioblastoma cells through ERK signaling, contributing to the self-regulatory machinery of CXCL12.

We investigated the impact of this regulation in glioblastoma cells on macrophages. Consistently, the MLC proliferation and the expression of IL-6 and IL1R1 were promoted by incubation with conditioned medium from parental glioblastoma cells transfected with siCXCR7. However, co-transfection with siCXCR7-siCXCL12 reversed this effect (Supplementary Fig. S4A, B). Furthermore, culturing in conditioned medium from U87MG, A172, or Pt#3 cells transfected with siCXCR7 or GL261-shCXCR7 cells resulted in an increase in PD-L1 expression, which was diminished in conditioned medium from cells co-transfected with siCXCR7-siCXCL12 or GL261-shCXCR7 transfected with siCXCL12 (Fig. 3G, H, and Supplementary S4C, D). Taken together, these results suggested that CXCR7 functions as a negative regulator of CXCL12-mediated events.

### CXCR7 knockdown in glioblastoma cells promoted malignancy

**Fig. 4:**
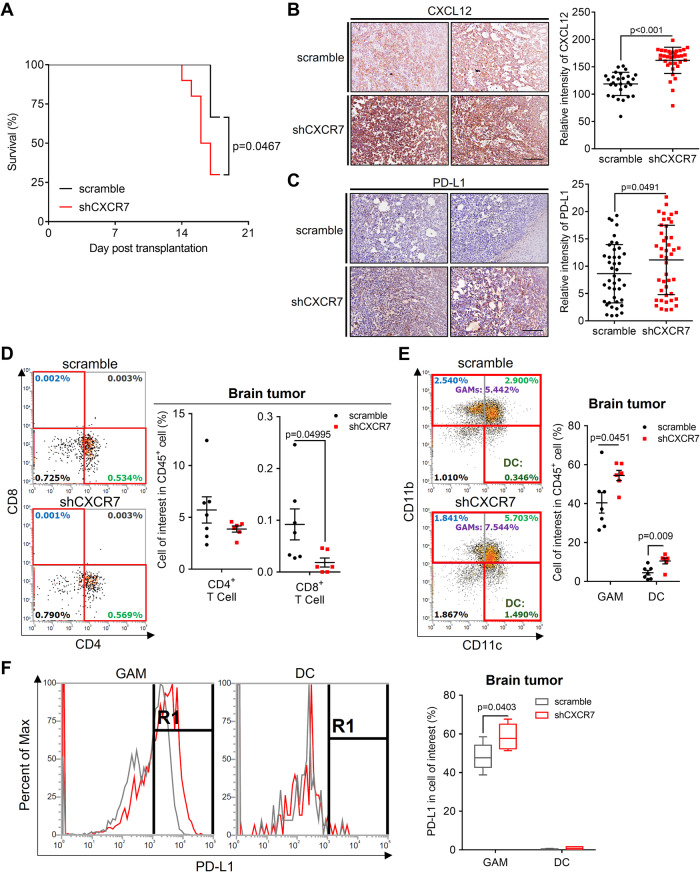
CXCR7 knockdown in glioblastoma cells conferred poor survival and the formation of immunosuppressive microenvironment. **A** Survival curve of GL261-bearing mouse with stably-expressing shRNA of scramble or CXCR7 in GL261 cells; *N* = 10 mice per groups; **B** IHC staining and histogram of CXCL12 expression; scale bar = 100 μm; **C** IHC staining and histogram of PD-L1 expression; scale bar = 100 μm; **D** Multicolor flow cytometry revealing the composition of CD4^+^ and CD8^+^ T cells in the GL261-bearing mouse brain tumors; **E** The composition of GAM and DC in the GL261-bearing mouse brain tumors; **F** PD-L1 expression in GAM and DC in GL261-bearing mouse brain tumors. Survival difference in **A** was compared using a log-rank (Mantel–Cox) test. Statistical differences in **B**–**F** were compared between the scramble and shCXCR7 groups by using an unpaired *t* test.

To assess the in vivo impact of these effects, GL261-shCXCR7 cells were applied to an orthotopic glioblastoma model. The survival curve revealed significantly worse outcomes for the shCXCR7 group than for the scramble-control group (median survival of 16.5 days and undefined, respectively; *N* = 10 mice/group; Fig. 4A). Supportively, the rapid weight loss was observed in the shCXCR7 group (Supplementary Fig. S5A). Optical images and H&E staining revealed larger intracranial tumors in the shCXCR7 group than in the scramble-control group (Supplementary Fig. S5B, C). Consistently, IHC analysis revealed enhanced CXCL12 and PD-L1 expression in the shCXCR7 group (Fig. 4B, C). Furthermore, flow cytometry analysis of the tumor samples revealed that although the proportion of CD45^+^, CD45^+^/CD19^+^, CD45^+^/CD3^+^, and CD45^+^/CD3^+^/CD4^+^ cells did not significantly differ, the proportion of CD45^+^/CD3^+^/CD8^+^ cells decreased in the shCXCR7 group compared with that in the scramble-control group (Supplementary Fig. S5D: gating strategy; Fig. 4D and Supplementary Fig. S5E, F for the data). Additionally, the proportion of GAMs and dendritic cells (DCs) increased (Fig. 4E). Notably, in the shCXCR7 group, higher PD-L1 expression was observed in GAMs, but not in other cells (Fig. 4F and Supplementary Fig. S5G). Otherwise, GL261-shCXCR7 cells did not affect the immune profiles of the spleen, bone marrow, or peripheral blood (Supplementary Fig. S5H–J). Thus, the orthotopic model of GL261-shCXCR7 cells exhibited worse survival, accompanied by increased CXCL12 and PD-L1 expression in GAMs, and a decreased proportion of CD8^+^ T cells.

### VUF11207 restored GAM-suppressed T-cell functions

**Fig. 5:**
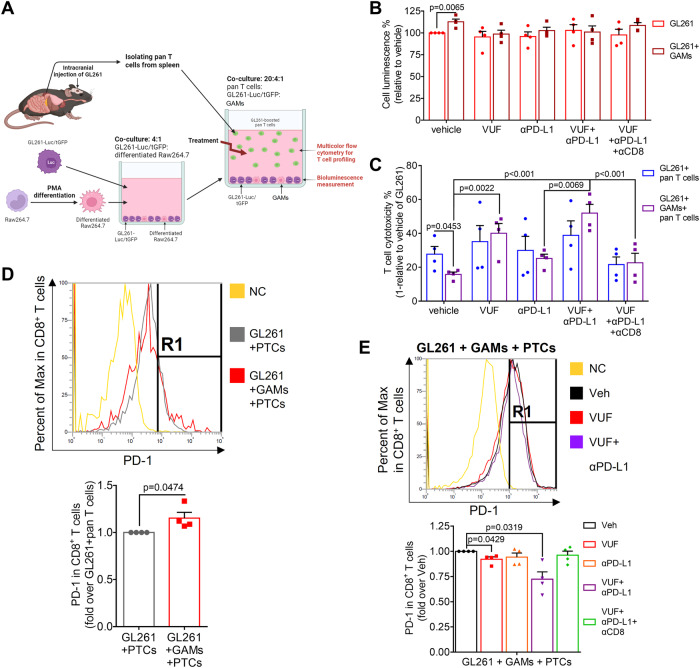
CXCR7 activation restored GAM-impaired T cell cytotoxicity and reversed GAM-induced T cell exhaustion. **A** Experiment scheme. Mouse Raw264.7 cells were differentiated into MLCs by PMA. GAMs were induced by co-culturing the MLCs with luciferase/turboGFP-expressing GL261 (GL261-Luc/tGFP) cells. PTCs were isolated from the spleen of GL261-boosted mice. Cells were treated with PBS/10 μg/ml IgG/10 μg/ml αHRPN as control, 500 nM VUF, 10 μg/ml αPD-L1, 10 μg/ml αCD8β, or the combination treatments; **B** Cell luminescence of GL261-Luc/tGFP cells in the absence or the presence of GAMs under treatments; **C** T cell cytotoxicity of PTCs co-cultured with GL261-Luc/tGFP cells in the absence or the presence of GAMs under treatments; **D** multicolor flow cytometry revealing the PD-1 expression in CD8^+^ T cells of PTCs co-cultured with GL261-Luc/tGFP cells in the absence or the presence of GAMs; **E** PD-1 expression in CD8^+^ T cells of PTCs co-cultured with GL261-Luc/tGFP cells and GAMs in the presence of treatments. Statistical differences in **B**, **C** and **E** were assessed using one-way ANOVA. PD-1 expression in **D** was compared between in the absence and the presence of GAMs by using an unpaired *t* test.

We implemented a co-culture system to evaluate the impact of VUF and anti-PD-L1 antibody (αPD-L1) on the T-cell functions and tumor-cell growth (Fig. 5A). The results revealed that GAMs promoted the growth of GL261 cells, but this effect was nullified after co-incubation with VUF alone and VUF-αPD-L1 combination (Fig. 5B). Additionally, a transwell-based co-culture system with ATP-independent MTT assay was used to confirm these phenomena (Supplementary Fig. S6A). The results showed that co-culturing with GAMs facilitated cell growth of Pt#3 and GL261 cells whereas this effect was eliminated after co-incubation with VUF alone and VUF-αPD-L1 combination (Supplementary Fig. S6B). Subsequently, in the co-culture system with added ex vivo PTCs, T-cell cytotoxicity against GL261 cells was suppressed in the presence of GAMs (Fig. 5C). The cytotoxic effect of PTCs was restored after co-incubation with VUF alone and VUF-αPD-L1 combination, but not after incubation with αPD-L1 alone. Moreover, depletion of CD8^+^ T cells by αCD8β mitigated the effects of the VUF-αPD-L1 combination (Fig. 5C). Flow cytometry analysis revealed that compared with PTCs co-cultured with GL261 cells, PTCs co-cultured with GL261 cells and GAMs exhibited the increased PD-1 expression in CD8^+^ T cells (Supplementary Fig. S6C: gating strategy; Fig. 5D). Notably, VUF, αPD-L1, and VUF-αPD-L1 combination did not directly affect PD-1 expression in CD8^+^ T cells (Supplementary Fig. S6D). Co-incubation with VUF alone and VUF-αPD-L1 combination reduced PD-1 expression in CD8^+^ T cells in PTCs co-cultured with GL261 cells (Supplementary Fig. S6E) and in PTCs co-cultured with GL261 cells and additional GAMs (Fig. 5E). Together, these findings revealed that VUF restored the anti-tumor functions of T cells suppressed by GAMs.

### VUF11207 evoked the anti-tumor effect of αPD-L1

**Fig. 6:**
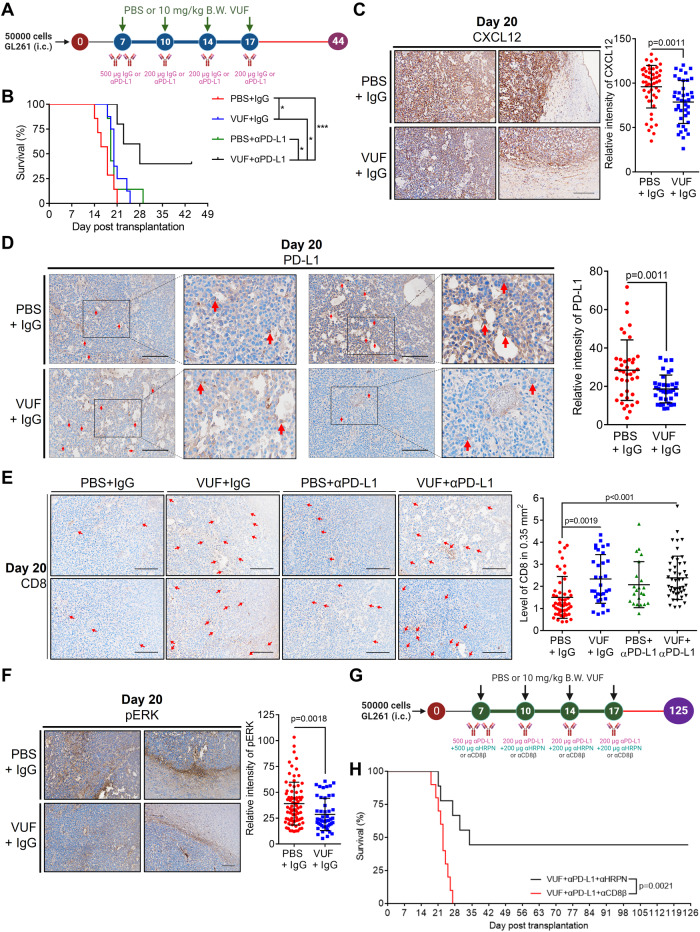
CXCR7 agonist prolonged survival and sensitized glioblastoma to PD-L1 antibodies. **A** Experiment scheme. GL261-bearing mice were treated with PBS/IgG as control, 10 mg/kg VUF, αPD-L1, or the combination treatments; *N* = 10 mice per groups; **B** Survival curve of treated GL261-bearing mice; **C** IHC staining and histogram of CXCL12 expression in PBS/IgG control and VUF/IgG; scale bar = 100 μm; **D** IHC staining and histogram of PD-L1 expression in PBS/IgG control and VUF/IgG; scale bar = 100 μm; **E** IHC staining and histogram of CD8 level in PBS/IgG control, VUF/IgG, αPD-L1, and the combination treatments; scale bar = 100 μm; **F** IHC staining for phosphorylated ERK in the treated GL261-bearing mouse brains on postinjection day 20; **G** Experiment scheme. αCD8β was used to deplete CD8^+^ T cells. GL261-bearing mice were treated with VUF/αPD-L1/αHRPN as control or VUF/αPD-L1/αCD8β; *N* = 10 mice per groups; **H** Survival curve of treated GL261-bearing mice. Red arrows in **D** indicate PD-L1 expressing macrophages. Red arrows in **E** indicate CD8^+^ T cells. Survival in **B** and **H** was compared using a log-rank (Mantel–Cox) test. IHC staining in **C**, **D** and **F** was compared using an unpaired *t* test. Statistical differences in **E** were assessed using one-way ANOVA.

We investigated the therapeutic potential of CXCR7 activation by administering VUF, with or without αPD-L1, in an orthotopic mouse model (Fig. 6A). The VUF-αPD-L1 combination significantly prolonged survival compared with control, VUF, or αPD-L1 monotherapy (median survival of 28, 18, 20, and 19 days, respectively; *N* = 10 mice/group; Fig. 6B). Notably, all groups, except the VUF-αPD-L1 combination group, exhibited weight loss within 20 days after tumor implantation (Supplementary Fig. S7A). Tissue staining revealed that the tumor sizes on post-implantation day 20 in the VUF monotherapy and VUF-αPD-L1 combination groups were smaller than those in the control group (Supplementary Fig. S7B). IHC staining revealed that VUF monotherapy reduced CXCL12 and PD-L1 expression, particularly PD-L1 in the GAMs (Fig. 6C, D). Moreover, compared to the control groups, the proportion of CD8^+^ T cells increased in the tumor tissues of VUF monotherapy and the VUF-αPD-L1 combination groups (Fig. 6E). Furthermore, IHC staining demonstrated that VUF monotherapy attenuated phosphorylated ERK expression (Fig. 6F). Notably, on post-implantation day 44, two mice from the VUF-αPD-L1 combination group survived; one exhibited a tumor-free brain and the other mouse had a small tumor in the brain enriched with CD8^+^ T cells (Supplementary Fig. S7C, D). Subsequently, αCD8β was administered to elucidate the role of T cells in the combination therapy (*N* = 10 mice/group; Fig. 6G). The results revealed that 4 of 9 mice receiving VUF-αPD-L1-αHRPN combination in the first 17 days achieved long-term survival, whereas all mice receiving VUF-αPD-L1-αCD8β combination succumbed within 27 days after tumor implantation (Fig. 6H). Increased body weight in the VUF-αPD-L1-αHRPN combination group supported treatment efficiency (Supplementary Fig. S7E). Taken together, these studies suggested that CXCR7 activation sensitizes glioblastoma cells to ICBs, leading to significant anti-tumor effects.

## Discussion

With advances in the understanding of the pathophysiology of brain tumors, various investigational strategies have been developed to target GAMs [29]. Herein, we reported an association between increased CXCL12 expression and disease progression (Fig. 1 and Supplementary Fig. S3A, B). GAMs can be activated by tumor-derived CXCL12, which is negatively regulated by CXCR7 (Figs. 2 and 3G, Supplementary Figs. S2 and S4). This observation suggests that CXCL12 acts as a communicator for tumors and thus shapes the TME. When exposed to exogenous CXCL12^hi^ or conditioned medium from CXCR7-knockdown tumor cells, MLCs exhibited upregulated pro-tumorigenic gene expression (Supplementary Figs. S2B, C and S4B), indicating a correlation between these factors and poor outcomes in glioblastoma [30, 31]. Accordingly, enhanced GAM-related profiles in CXCR7-knockdown tumors resulted in worse survival rates (Fig. 4). In terms of IC effect, CXCL12 increased PD-L1 expression in GAMs through the NF-κB pathway (Fig. 2). Furthermore, GAMs inhibited the T-cell functions (Fig. 5C, D), which was consistent with the contribution of GAM-associated PD-L1 to immunosuppression in glioblastoma [4]. Notably, this effect was reversed by VUF, demonstrating the restoration of cytotoxicity and suppression of PD-1 expression in T cells (Fig. 5C, E, and Supplementary Fig. S6C) [32]. Our study provides a novel perspective for targeting CXCR7-CXCL12 to inhibit pro-tumorigenic immunity.

Selective inhibitors targeting CXCL12/CXCR4, such as plerixafor and BL-8040, have been investigated for their anti-tumor activities, showing variable effects in various cancers [33, 34]. CXCR7 agonists can be alternative choices but with the diverse roles of CXCR7 have been reported, ranging from compensating for CXCR4 inhibitors, promoting cancer progression, treatment failure [34, 35], and to counteracting CXCL12 function [35, 36]. Hereby, we identified an inverse correlation between CXCR7 and CXCL12 expression in glioblastoma cells (Fig. 3B, C, Supplementary Fig. S3A, C, D). Mechanistically, CXCL12 likely activates ERK via CXCR4 [37]. ERK activation triggers the feedback loop of CXCL12 [28]. Our data showed that CXCR7 knockdown-induced CXCL12 was reversed by ERKi (Fig. 3D, E). Notably, VUF was developed to counteract CXCL12-induced events [23], and prevents the initiation of ERK1/2 phosphorylation [38]. VUF inhibited CXCL12-activated pERK in vitro and in vivo (Figs. 3F and 6F), strengthening the compensatory role of CXCR7 in self-regulation with that reported by Nugraha [23]. However, we cannot rule out other possibilities, such as acting as a scavenger to sequester CXCL12 [36, 39].

VUF also demonstrated a significant survival benefit in mice with glioblastoma, consistent with the treatment effect observed with CXCL12 inhibitors (Fig. 6B). Notably, VUF, exogenous CXCL12^hi^, CXCR7 knockdown, or CXCR7 overexpression had a minimal and inconsistent impacts on tumor cell and CXCR4 expression (Supplementary Fig. S3C, E, F, I–L). This targeted-activation strategy may have a prominent advantage in potentiating CD8^+^ T-cell functions, particularly because treatment with αCD8β abrogated the benefits of the treatment under investigation (Fig. 6G). Interestingly, given that CXCR7 have fundamental roles in cancer cells, Walters, et al., discovered a conversed strategy with inhibiting CXCR7 to mitigate the post-irradiation recurrence to prolong the survival of brain-tumor carrying animal model [18]. In our study, we noted that CXCR7 agonist VUF yielded prolonged survival outcome by overcoming ICB resistant mechanism. This highlighted the CXCL12-mediated cell-cell communication, which made our study different to the CXCR7-related intrinsic cellular reaction under irradiation stimuli in glioblastoma. As thus, the advantage of activating or inhibiting CXCR7 activity for treatment would largely depend on conditions.

Glioblastoma is characterized as an immunoprivileged disease [40], featuring low T-cell infiltration into the tumor tissue (Figs. 1E, 4D and 6E). Enhanced T-cell cytotoxicity and infiltration, and reduced PD-1 expression in CD8^+^ T cells with VUF treatment are highly valuable (Fig. 5E, Supplementary Fig. S6C, E). Excessive CXCL12 in the TME can impede T-cell migration [41]. Supportively, liposomal-plerixafor has been reported to enhance T-cell infiltration in a triple-negative breast cancer model [42]. Given the advantage of blocking the CXCL12 effect, CXCL12/CXCR4 inhibitors have shown to enhance the therapeutic potential of αPD-1 against tumors [43, 44]. NOX-A12, a novel CXCL12 inhibitor, enhances T-cell infiltration, thereby improving the effectiveness of αPD-1 in a mouse CT26 model [45]. Novel strategies, such as bifunctional small molecules targeting CXCL12 and PD-L1 simultaneously, are also under development [46]. Our data revealed that VUF-αPD-L1 combination achieved a long-term survival in 40% of the mice after treatment discontinuation (Fig. 6B, H). Encouragingly, examination of the brains revealed free or only small tumors with enriched infiltration of CD8^+^ T cells in mice receiving the VUF-αPD-L1 combination (Supplementary Fig. S7C, D). Supportively, Wu et al. reported that αCXCR4/αPD-1 treatment in a glioblastoma mouse model resulted in long-term survival [47]. Considering that CXCR4 is expressed in a broad range of immunocytes (Fig. 3A), fundamental functions such as maintaining CD8^+^ memory T-cell homeostatic self-renewal may be affected by CXCR4 inhibitors [48]. This can be neglected by targeting CXCR7 because of lacking detection in CD8^+^ T cells [49] and the distribution in tumor cells (Fig. 3A). Our strategy provides the advantages of avoiding CXCR4-related adverse effects and directly targeting tumor cells, thus having the potential to harmonize the anti-tumor effect of ICBs.

**Fig. 7:**
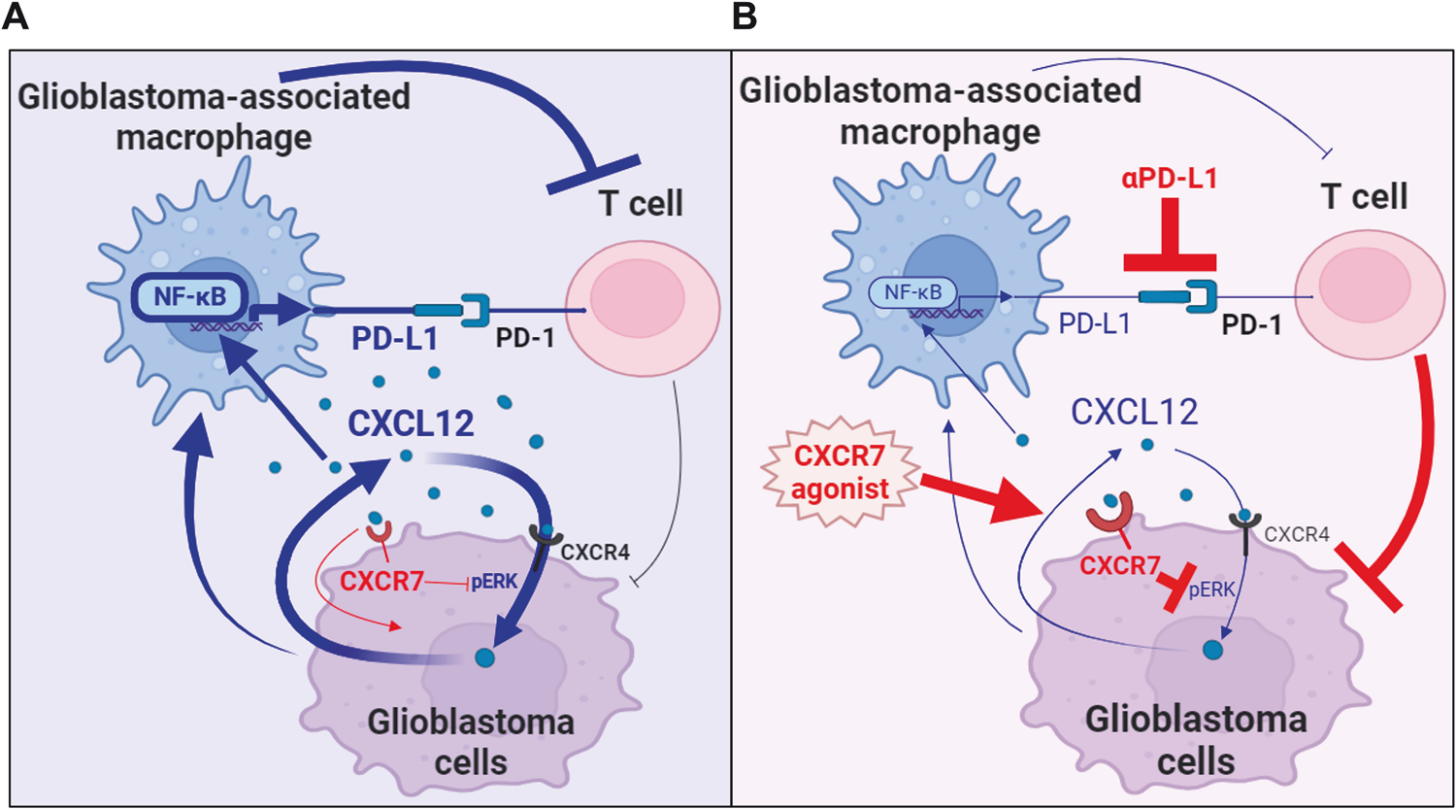
The summary of the effects of CXCR7-CXCL12 regulatory loop on glioblastoma cells and its microenvironment. **A** Reduced CXCR7 expression in glioblastoma cells resulted in the accumulation of CXCL12. This increased PD-L1 expression in GAMs, subsequently inhibited T-cell recruitment and functions. **B** By CXCR7 agonist, the anti-tumor immunity was retrieved through reversed CXCL12-mediated immunity. This sensitized glioblastoma to the αPD-L1 therapy. Note that the blue arrows indicate the intrinsic cell-cell interaction and the reactions of mediators among glioblastoma cells, GAMs, and T cells; the red arrows show the associated reaction responding to the stimuli of CXCR7 agonist.

In conclusion, our study revealed that CXCR7 downregulation in glioblastoma cells leads to the accumulation of CXCL12. This regulation contributes to increased PD-L1 expression in GAMs, reduced T-cell infiltration and functions. VUF elicited immunoreactions by reducing CXCL12-mediated GAM induction, enhancing CD8^+^ T-cell activity, and promoting their infiltration into the tumor tissue. This TME remodeling sensitized glioblastoma cells to αPD-L1 therapy (Fig. 7), thus provided a mechanistic basis for CXCR7 activation to enhance anti-tumor immunity that could support immunotherapies. CXCR7 agonizts such as VUF or other mediators are thus warranted for further exploration of their application in cancer treatment.

### Supplementary information

Supplementary Method

Supplementary Tables

Supplementary Figure Legends

Supplementary Figure S1-S7

Original data file

## Data Availability

The data for bulk RNA sequencing and scRNA seq in this study are available from Kwang-Yu Chang (kwang2@nhri.edu.tw) upon reasonable request.
