# Supplementary material for: CXCR7 activation evokes the anti-PD-L1 antibody against glioblastoma by remodeling CXCL12-mediated immunity
Source: Cell Death Dis. 2024 Jun 19;15(6):434. doi: 10.1038/s41419-024-06784-6 (PMC11187218; doi:10.1038/s41419-024-06784-6)
Supplement: Supplementary file 1 — Supplementary Method [file 41419_2024_6784_MOESM1_ESM.docx]

**Supplementary Method**

**HMC-3 and SC Cell culture**

Human microglia cell line HMC-3 and human primary peripheral blood mononuclear cells SC were purchased from the American Type Culture Collection (USA). HMC-3 cells were maintained in MEM medium (Gibco) supplemented with P/S (Gibco), and 10% heated-inactivated FBS (Hyclone). HMC-3 cells were incubated at 37℃ in humidified 95% air/5% CO_2_. SC cells were maintained in IMDM (Gibco) supplemented with P/S, 50 pM β-mercaptoethanol, nonessential amino acids (Gibco), and 10% FBS. The SC cells were incubated at 37℃ in humidified 92.5% air/7.5% CO_2_.

**Macrophage differentiation and polarization**

SC cells were differentiated into M0 macrophages by incubating them with 200 nM PMA (Sigma) for 72 hours. M0 macrophages and HMC-3 cells were treated with human or mouse CXCL12 (PeproTech). The mRNA expression profiles of M0- and CXCL12-treated macrophages were detected using reverse-transcription quantitative real-time polymerase chain reaction (qRT-PCR).

**Colony formation assay**

We seeded 400 cells/well in a 6-well plate, and VUF treatments were provided on the subsequent day. The media containing the VUF treatment were replaced after 4 days with fresh medium. The detailed procedures have been reported previously^21^.

**Plasmid-DNA-based gene modulation**

Lentiviruses carrying scramble (PCMV6XL6) or human CXCR7 (Sc112665) were purchased from OriGene Technologies, Inc. Cells were transduced with a lentiviral scramble vector or human CXCR7.

**Development of stably expressing shCXCR7 GL261 cells**

Lentiviruses carrying scramble (pLKO_TRC001) or shCXCR7 (TRCN0000026660, TRCN0000022177) vectors were purchased from the RNAi Core laboratory of Academia Sinica (Taiwan). GL261 cells were transduced with a lentiviral scramble vector or shCXCR7. To stably express the scramble vector or shCXCR7, puromycin selection (5 μg/mL; Cayman) was conducted. The selected cells were maintained in culture media with 1 μg/mL puromycin.

**Western blot analysis**

The proteins from the total cell lysate were extrated by RIPA buffer (BioRad), separated through SDS-PAGE, and transferred onto polyvinylidene difluoride membranes (Millipore). The membranes were blocked with 5% nonfat milk and incubated overnight with the primary antibodies (Table S3). They were then washed and incubated with secondary antibodies (Table S3). After eliciting signals with a chemiluminescence substrate with ECL (GE Healthcare), we detected protein expression intensity and quantified the density of each protein band using UVP software (Thermo-Fisher Scientific).

**Transwell-based co-culture system**

Human THP-1 cells or mouse Raw264.7 cells were differentiated into MLCs in upper chambers of transwell by using PMA for 72 hours. GAMs were induced by co-culturing MLCs with the human Pt#3 cells or mouse GL261 cells in the bottom of well for 1 day. The treatments were added in both upper chamber of transwell and lower chamber of well for 48 hours. MTT assay was performed for measuring tumor cell growth.
