# Supplementary material for: CXCR7 activation evokes the anti-PD-L1 antibody against glioblastoma by remodeling CXCL12-mediated immunity
Source: Cell Death Dis. 2024 Jun 19;15(6):434. doi: 10.1038/s41419-024-06784-6 (PMC11187218; doi:10.1038/s41419-024-06784-6)
Supplement: Supplementary file 2 — Supplementary Tables [file 41419_2024_6784_MOESM2_ESM.docx]

**Supplementary Tables**

**Table S1. Primer sequences for qRT-PCR**

| **Species** | **Name** | **Sequence (5' to 3') of qPCR primers** | |
| --- | --- | --- | --- |
|  |  | **Forward** | **Reverse** |
| **Human** | CXCL12 | ATT CTC AAC ACT CCA AAC TGT GC | ACT TTA GCT TCG GGT CAA TGC |
|  | CXCR4 | ACG CCA CCA ACA GTC AGA G | AGT CGG GAA TAG TCA GCA GGA |
|  | CXCR7 | TCT GCA TCT CTT CGA CTA CTC A | GTA GAG CAG GAC GCT TTT GTT |
|  | IL1β | ATG ATG GCT TAT TAC AGT GGC AA | GTC GGA GAT TCG TAG CTG GA |
|  | IL1R1 | ATG AAA TTG ATG TTC GTC CCT GT | ACC ACG CAA TAG TAA TGT CCT G |
|  | IL6 | ACT CAC CTC TTC AGA ACG AAT TG | CCA TCT TTG GAA GGT TCA GGT TG |
|  | MMP9 | GGG ACG CAG ACA TCG TCA TC | TCG TCA TCG TCG AAA TGG GC |
|  | MRC1 | GGG TTG CTA TCA CTC TCT ATG C | TTT CTT GTC TGT TGC CGT AGT T |
|  | IL1R2 | ATG TTG CGC TTG TAC GTG TTG | CCC GCT TGT AAT GCC TCC C |
|  | PD-L1 | GGA CAA GCA GTG ACC ATC AAG | CCC AGA ATT ACC AAG TGA GTC CT |
|  | GAPDH | TGC ACC ACC AAC TGC TTA GC | GGC ATG GAC TGT GGT CAT GAC |
| **Mouse** | CXCL12 | TGC ATC AGT GAC GGT AAA CCA | CAC AGT TTG GAG TGT TGA GGA T |
|  | CXCR4 | GAC TGG CAT AGT CGG CAA TG | AGA AGG GGA GTG TGA TGA CAA A |
|  | CXCR7 | AGC CTG GCA ACT ACT CTG ACA | GAA GCA CGT TCT TGT TAG GCA |
|  | IL1β | GCA ACT GTT CCT GAA CTC AAC T | ATC TTT TGG GGT CCG TCA ACT |
|  | IL1R1 | GTG CTA CTG GGG CTC ATT TGT | GGA GTA AGA GGA CAC TTG CGA AT |
|  | IL6 | TGG GGC TCT TCA AAA GCT CC | AGG AAC TAT CAC CGG ATC TTC AA |
|  | MMP9 | CTG GAC AGC CAG ACA CTA AAG | CTC GCG GCA AGT CTT CAG AG |
|  | MRC1 | CTC TGT TCA GCT ATT GGA CGC | CGG AAT TTC TGG GAT TCA GCT TC |
|  | IL1R2 | GTT TCT GCT TTC ACC ACT CCA | GAG TCC AAT TTA CTC CAG GTC AG |
|  | PD-L1 | GCT CCA AAG GAC TTG TAC GTG | TGA TCT GAA GGG CAG CAT TTC |
|  | GAPDH | AAC TTT GGC ATT GTG GAA GG | ACA CAT TGG GGG TAG GAA CA |

**Table S2. Treatment and chemicals**

| **Chemicals** | **Manufacturer** | **Cat#** |
| --- | --- | --- |
| Phorbol 12-Myristate 13-Acetate (PMA) | Sigma | P1585 |
| Lipopolysaccharides (LPS) | Sigma | L3129 |
| Recombinant human IFN | Peprotech | #300-02 |
| Recombinant human IL-4 | Peprotech | #200-04 |
| Recombinant mouse IFN | Peprotech | #315-05 |
| Recombinant mouse IL-4 | Peprotech | #214-14 |
| Recombinant human CXCL12 | Peprotech | #300-28A |
| Recombinant mouse CXCL12 | Peprotech | #250-20A |
| Recombinant mouse IL-2 | Peprotech | # 212-12 |
| Hygromycin B | Cayman | #14291 |
| Puromycin | Cayman | #13884 |
| Temozolomide (TMZ) | Sigma | T2577 |
| control siRNA-A | Santa Cruz | sc-37007 |
| CXCL12 siRNA (siCXCL12)-human | Santa Cruz | sc-39367 |
| CXCL12 siRNA (siCXCL12)-mouse | Santa Cruz | sc-39368 |
| CXCR7 siRNA (siCXCR7)-human | Santa Cruz | sc-95473 |
| CXCR7 siRNA (siCXCR7)-mouse | Santa Cruz | sc-142643 |
| VUF11207 | MedChemExpress | HY-110318 |
| αPD-L1 | BioXCell | BE0101 |
| Isotype IgG | BioXCell | BE0090 |
| αCD8β | BioXCell | BE0223 |
| αHRPN | BioXCell | BE0088 |
|  |  |  |

**Table S3. Information of antibodies**

| **Antibodies for multicolor flow cytometry** | **Manufacturer** | **Cat#** | **Dilution** |
| --- | --- | --- | --- |
| CD4-FITC | eBioscience | 11-0042-81 | 1:400 |
| CD8-PerCP_Cy5.5 | Biolegend | 100733 | 1:200 |
| CD11c-APC | BD | 550261 | 1:200 |
| PD-L1-AF680 | Bioss | bs-4941R | 1:100 |
| PD-1-BV711 | BD | 744547 | 1:50 |
| CD11b-APC-Cy7 | BD | 557657 | 1:100 |
| CD45-BV510 | Biolegend | 103138 | 1:400 |
| CD19-BV605 | BD | 563148 | 1:200 |
| CD3-PE-CF594 | BD | 562286 | 1:200 |
| **Antibodies for Western blotting** | **Manufacturer** | **Cat#** | **Dilution** |
| CXCR7 | GeneTex | GTX100027 | 1:750 |
| CXCR4 | Abcam | ab124824 | 1:1000 |
| ERK | Cell Signaling | #9102 | 1:1500 |
| pERK | Cell Signaling | #4377 | 1:1500 |
| beta-actin | Merck | MAB1501 | 1:10000 |
| **HRP-conjugated secondary antibodies** | | | |
| Goat anti-mouse | Merck | AP124P | 1:5000 |
| Goat anti-Rabbit | Merck | AP132P | 1:5000 |
| **Antibodies for Immunohistochemistry staining** | **Manufacturer** | **Cat#** | **Dilution** |
| CXCL12 | R&D | MAB350 | 1:100 |
| PD-L1 | R&D | AF1019 | 1:100 |
| CD8a | Invitrogen | 14-0808-80 | 1:100 |
