# Supplementary material for: CXCR7 activation evokes the anti-PD-L1 antibody against glioblastoma by remodeling CXCL12-mediated immunity
Source: Cell Death Dis. 2024 Jun 19;15(6):434. doi: 10.1038/s41419-024-06784-6 (PMC11187218; doi:10.1038/s41419-024-06784-6)
Supplement: Supplementary file 3 — Supplementary Figure Legends [file 41419_2024_6784_MOESM3_ESM.docx]

**Supplementary Figure Legends**

**Figure S1. The cell annotation of scRNA-seq dataset of naïve glioblastoma specimens and the correlation of CXCL12 and PD-L1/CD274 in our in-house transcriptomic dataset.**

**A.** The heatmap of the top-10 upregulated genes within each clusters; **B.** the correlation of CXCL12 and PD-L1 in the bulk RNA sequencing of 10 paired naïve-recurrent specimens from glioblastoma patients. The correlation of CXCL12 and PD-L1 was analyzed by the Pearson correlation coefficient.

**Figure S2. Glioblastoma-derived CXCR7-CXCL12 activated microglia and macrophages to become GAMs**

**A.** Macrophage proliferation of differentiated human THP-1, SC, and mouse Raw264.7 cells in the presence of exogenous CXCL12; **B.** mRNA expression of protumor markers (IL-1β, IL1R1, IL-6, MMP9, IL1R2, and MRC1) in differentiated THP-1 cells in the presence of exogenous CXCL12; **C.** mRNA expression of protumor markers (IL-6, IL1R1, MMP9, and IL1R2) in differentiated Raw264.7 cells in the presence of exogenous CXCL12; **D.** mRNA expression of PD-L1 in differentiated SC cells, and human microglia HMC-3 cells in the presence of exogenous CXCL12; **E.** the protein expression of IκB, phosphorylated and total NF-κB p65 in the presence of CXCL12 and/or NF-κBi. Statistical differences in Fig. S2A-C were generated using one-way ANOVA Statistical differences in Fig. S2D were compared using an unpaired *t* test.

**Figure S3. CXCR7 regulated the expression of CXCL12 in a growth-independent manner.**

**A.** mRNA expression of CXCL12, CXCR4, and CXCR7 in parental and temozolomide-resistant human glioblastoma cell lines U87MG and A172, and patient-derived glioblastoma cells Pt#3; **B.** the protein expression of CXCR4 and CXCR7 in parental and temozolomide-resistant U87MG cells; **C.** mRNA expression of CXCL12, CXCR4, and CXCR7 in parental glioblastoma cells with siRNAs of CXCR7; **D.** mRNA expression of CXCL12, CXCR4, and CXCR7 in GL261 cells stably-expressing shRNA of scramble or CXCR7-26660 or CXCR7-22177; N=2; GL261 cells stably-expressing shRNA of CXCR7-26660 was selected for further study; **E.** mRNA expression of CXCL12, CXCR4, and CXCR7 in temozolomide-resistant glioblastoma cells with CXCR7 overexpression; **F.** mRNA expression of CXCL12, CXCR4, and CXCR7 in parental glioblastoma cells in the presence of VUF; **G.** mRNA expression of CXCL12 in the presence of CXCL12 and/or ERK inhibitor U0126; **H.** protein expression level of phosphorylated and total ERK in the presence of CXCL12hi and/or 500 nM VUF; **I.** MTT assay demonstrating cell viability of parental glioblastoma cells in the presence of CXCL12; **J.** MTT assay demonstrating cell viability of temozolomide-resistant human glioblastoma cells with CXCR7 overexpression; **K.** MTT assay demonstrating cell viability of parental glioblastoma cells in the presence of siCXCR7 or shCXCR7 alone or with siCXCL12; **L.** MTT assay demonstrating cell viability of parental glioblastoma cells in the presence of VUF. Statistical differences in Fig. S3A, S3C, and S3E were compared using an unpaired *t* test. Statistical differences in Fig. S3F-H and S3K were generated using one-way ANOVA.

**Figure S4. CXCR7 knockdown in glioblastoma cells induced GAMs and PD-L1 expression in GAMs via CXCL12.**

**A.** mRNA expression of PD-L1 in differentiated THP-1 cells and Raw264.7 cells with the conditioned medium of parental glioblastoma cells with siRNAs of CXCR7 and CXCL12; **B.** mRNA expression of IL6 and IL1R1 in differentiated THP-1 cells with the conditioned medium of parental glioblastoma cells with siRNAs of CXCR7 and CXCL12; **C.** mRNA expression of PD-L1 in differentiated THP-1 cells with the conditioned medium of parental glioblastoma cells with siRNAs of CXCR7 and CXCL12; **D.** mRNA expression of PD-L1 in human microglia cells HMC-3 with the conditioned medium of parental glioblastoma cells with siRNAs of CXCR7 and CXCL12. Statistical differences in Fig. S4A-D were compared using one-way ANOVA.

**Figure S5. The effects of CXCR7 knockdown in glioblastoma cells on tumor growth and immune cell profiling in brain tumors and systemic organs.**

**A.** Body weight changes in GL261-bearing mice; **B.** the images of GL261-bearing mouse brains; **C.** H&E staining images of the GL261-bearing mouse brains; **D.** gating strategy of multicolor flow cytometry for immune profiling in GL261-bearing mouse tissues; **E.** multicolor flow cytometry revealing the proportion of immune and non-immune cells in GL261-bearing mouse brain tumor tissues; **F.** the proportions of CD19^+^ B cells and CD3^+^ pan T cells in GL261-bearing mouse brain tumors; **G.** PD-L1 expression in immune and non-immune cells in GL261-bearing mouse brain tumors; **H.** the immune profiling in GL261-bearing mouse spleens; **I.** the immune profiling in GL261-bearing mouse bone marrows; **J.** the immune profiling in GL261-bearing mouse peripheral blood.

**Figure S6. CXCR7 activation by VUF11207 reduced PD-1 expression on GL261-associated T cells.**

**A.** The schematic of a transwell-based co-culture system; **B.** MTT assay demonstrating the cell viability of Pt#3 or GL261 cells in the absence or the presence of GAMs under treatments; **C.** Gating strategy of multicolor flow cytometry for T cell profiling; **D.** multicolor flow cytometry revealing the PD-1 expression in CD8^+^ T cells of pan T cells in the presence of treatments; **E.** the PD-1 expression in CD8^+^ T cells of pan T cells cocultured with GL261-Luc/tGFP cells in the presence of treatments. Statistical differences in Fig. S6B and S6E were assessed using one-way ANOVA.

**Figure S7. The effects of VUF-αPD-L1 combination on tumor size and body weight**

**A.** The body weight changes over time in the experiment shown in Fig. 6A; **B.** H&E staining images of the whole GL261-bearing brains on postinjection day 20; **C.** H&E staining images of whole GL261-bearing brains of combination treatment on postinjection day 44; **D.** IHC staining for CD8 in the treated GL261-bearing mouse brains on postinjection day 44; scale bar=100 μm; Red arrows indicate CD8^+^ T cells; **E.** the body weight changes over time in the experiment shown in Fig. 6F.
