# Supplementary material for: CXCR7 activation evokes the anti-PD-L1 antibody against glioblastoma by remodeling CXCL12-mediated immunity
Source: Cell Death Dis. 2024 Jun 19;15(6):434. doi: 10.1038/s41419-024-06784-6 (PMC11187218; doi:10.1038/s41419-024-06784-6)

Figure S1. The cell annotation of scRNA-seq dataset of naïve glioblastoma specimens and the correlation of CXCL12 and PD-L1/CD274 in our in-house transcriptomic dataset.

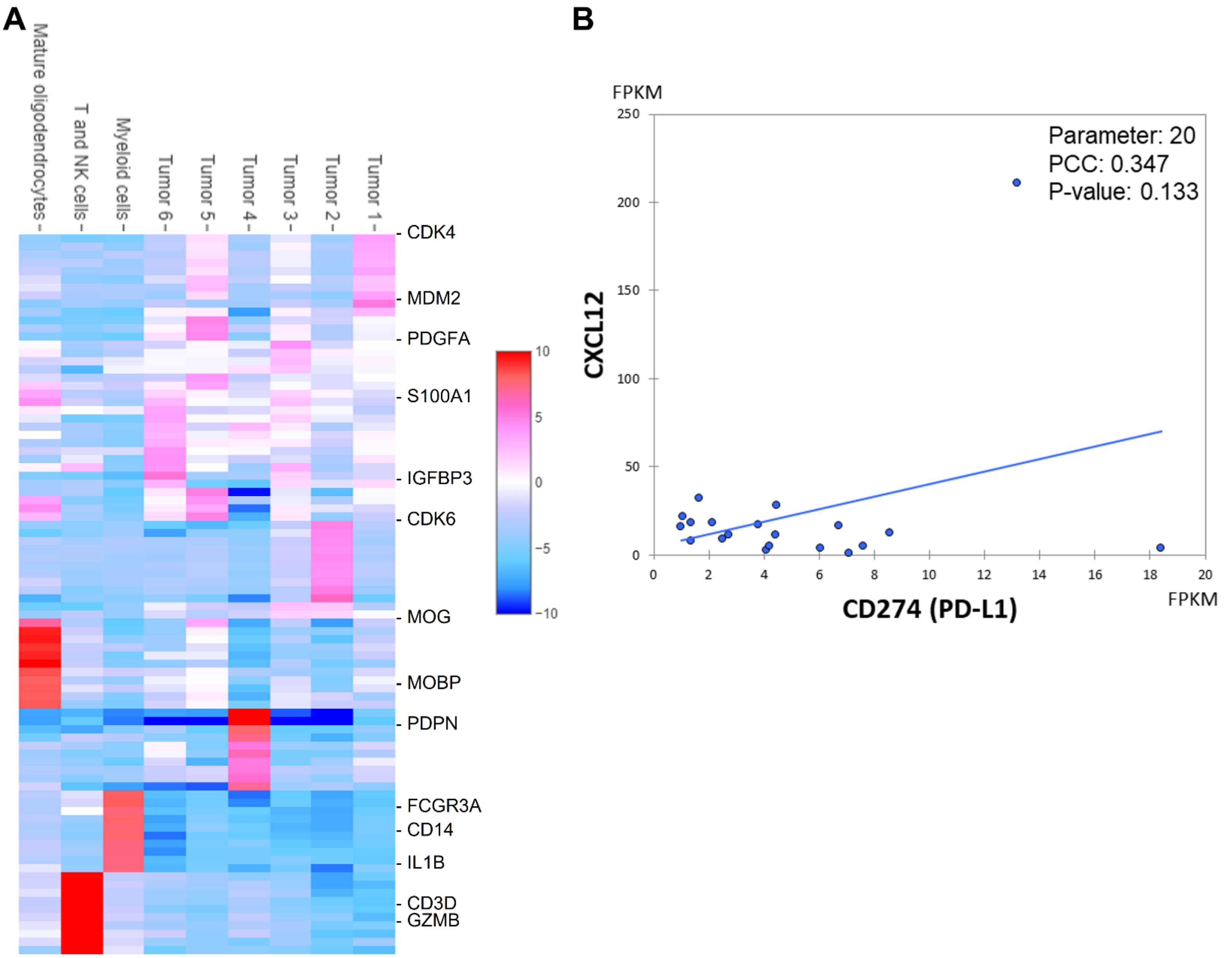

Figure S2. Glioblastoma-derived CXCR7-CXCL12 activated microglia and macrophages to become GAMs

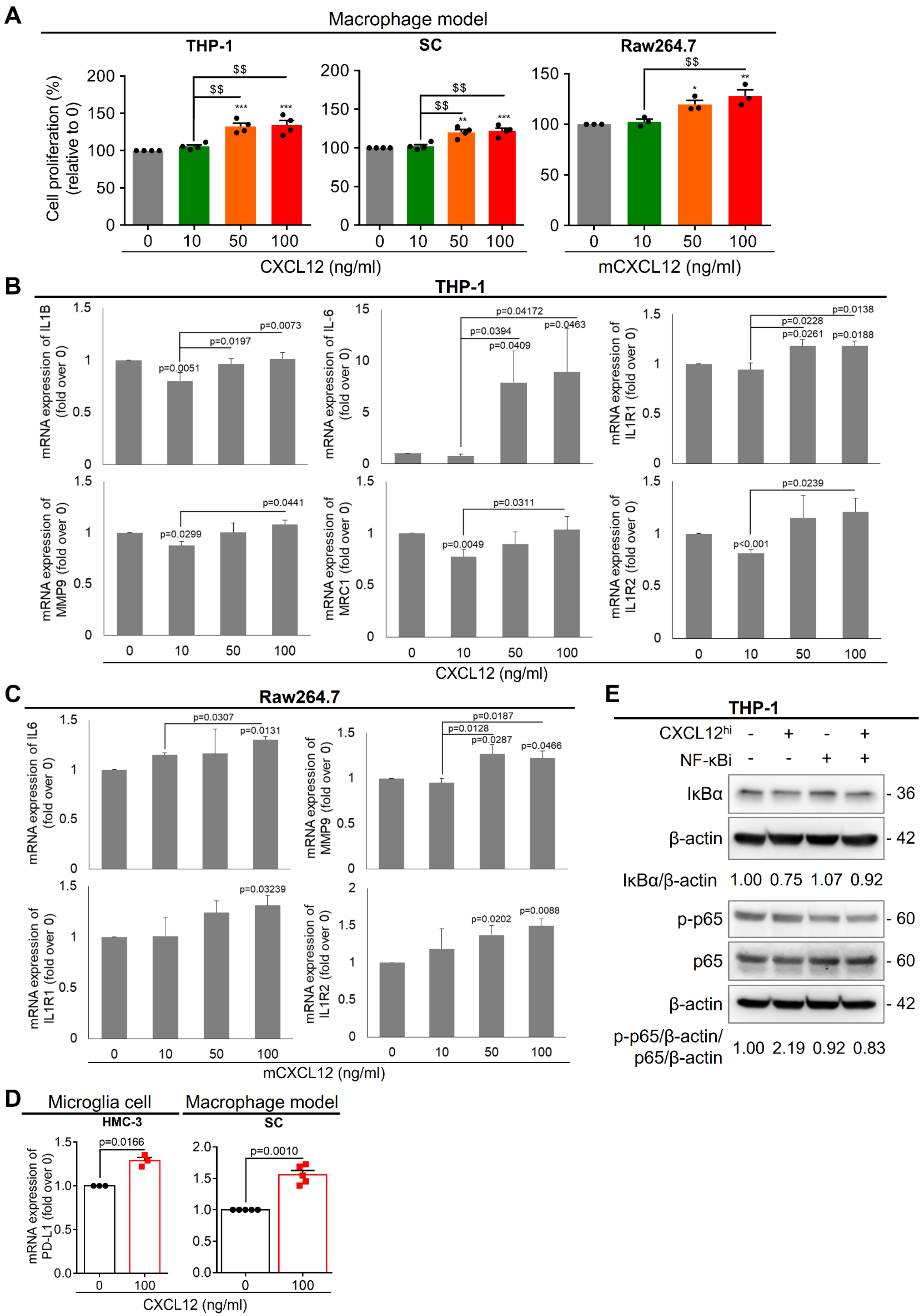

Figure S3. CXCR7 regulated the expression of CXCL12 in a growth-independent manner.

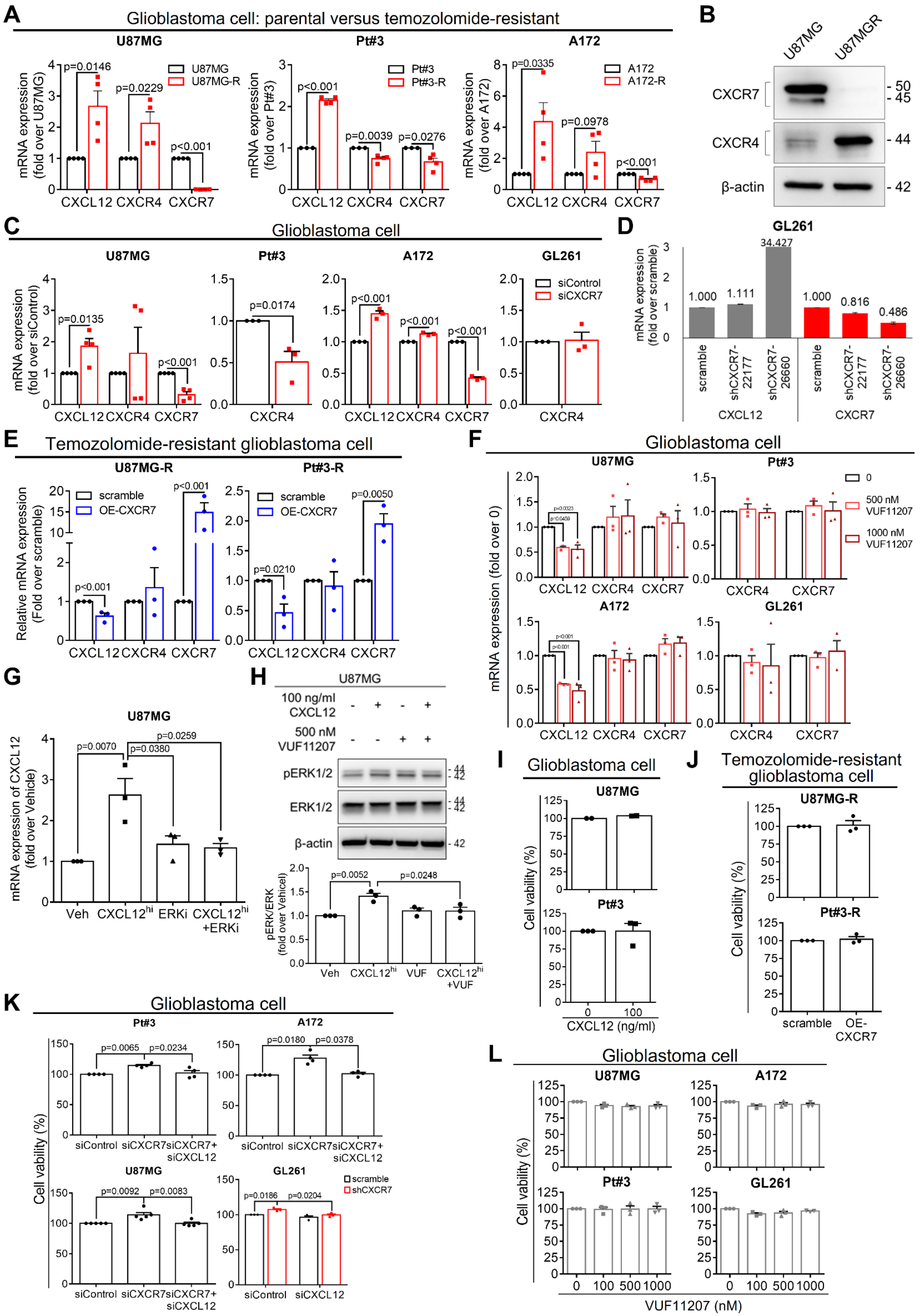

Figure S4. CXCR7 knockdown in glioblastoma cells induced GAMs and PD-L1 expression in GAMs via CXCL12.

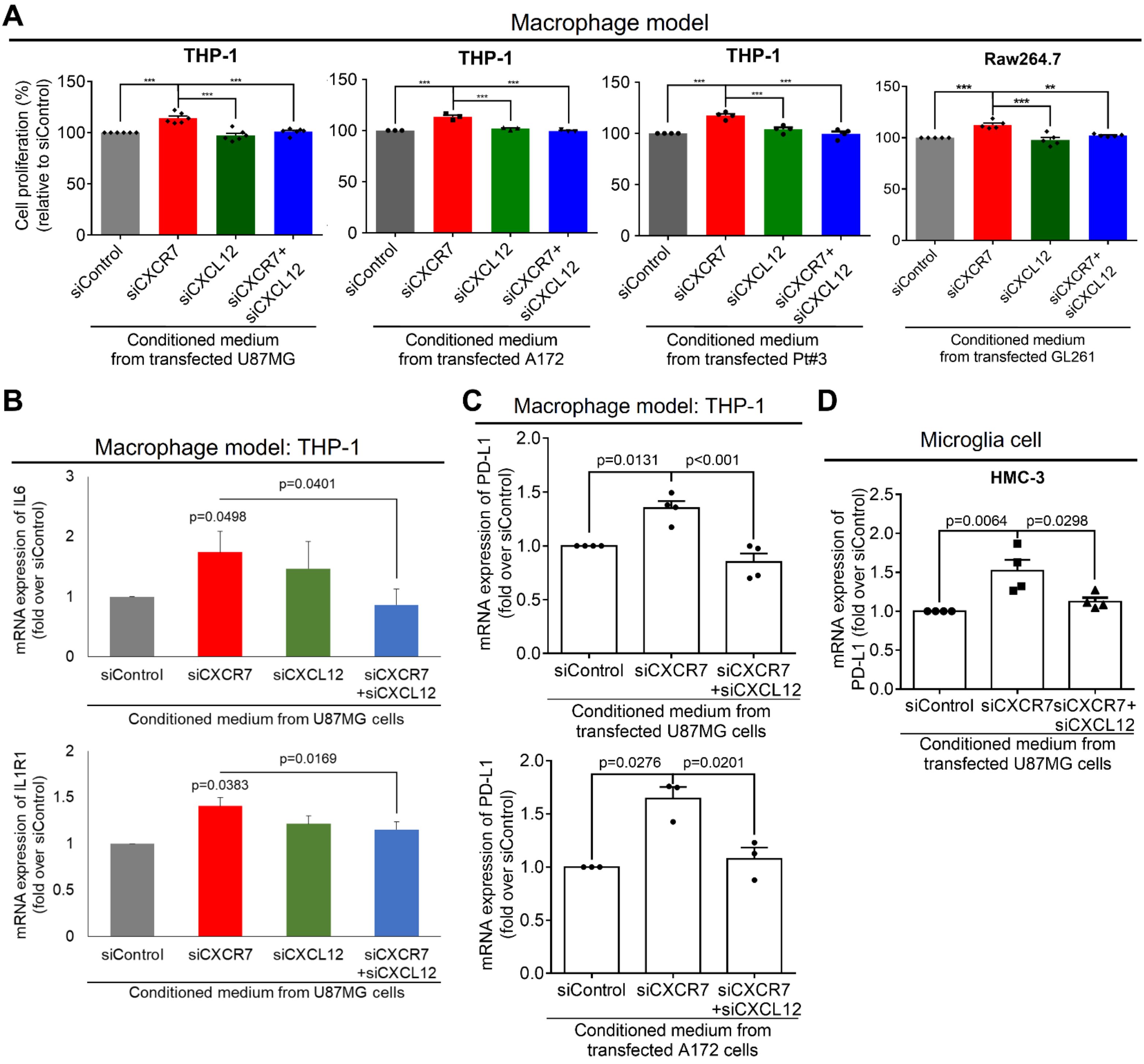

Figure S5. The effects of CXCR7 knockdown in glioblastoma cells on tumor growth and immune cell profiling in brain tumors and systemic organs.

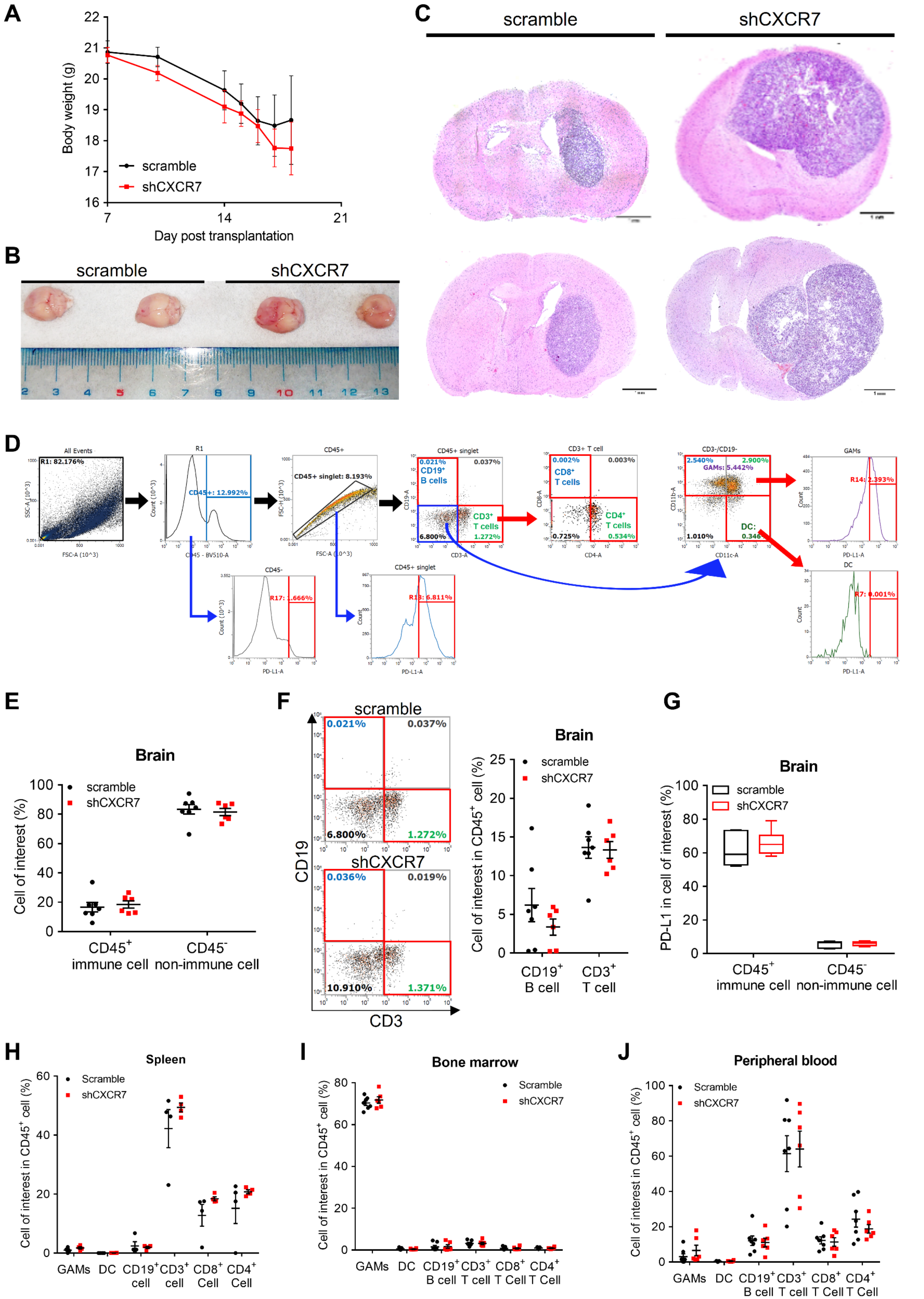

Figure S6. CXCR7 activation by VUF11207 reduced PD-1 expression on GL261-associated T cells

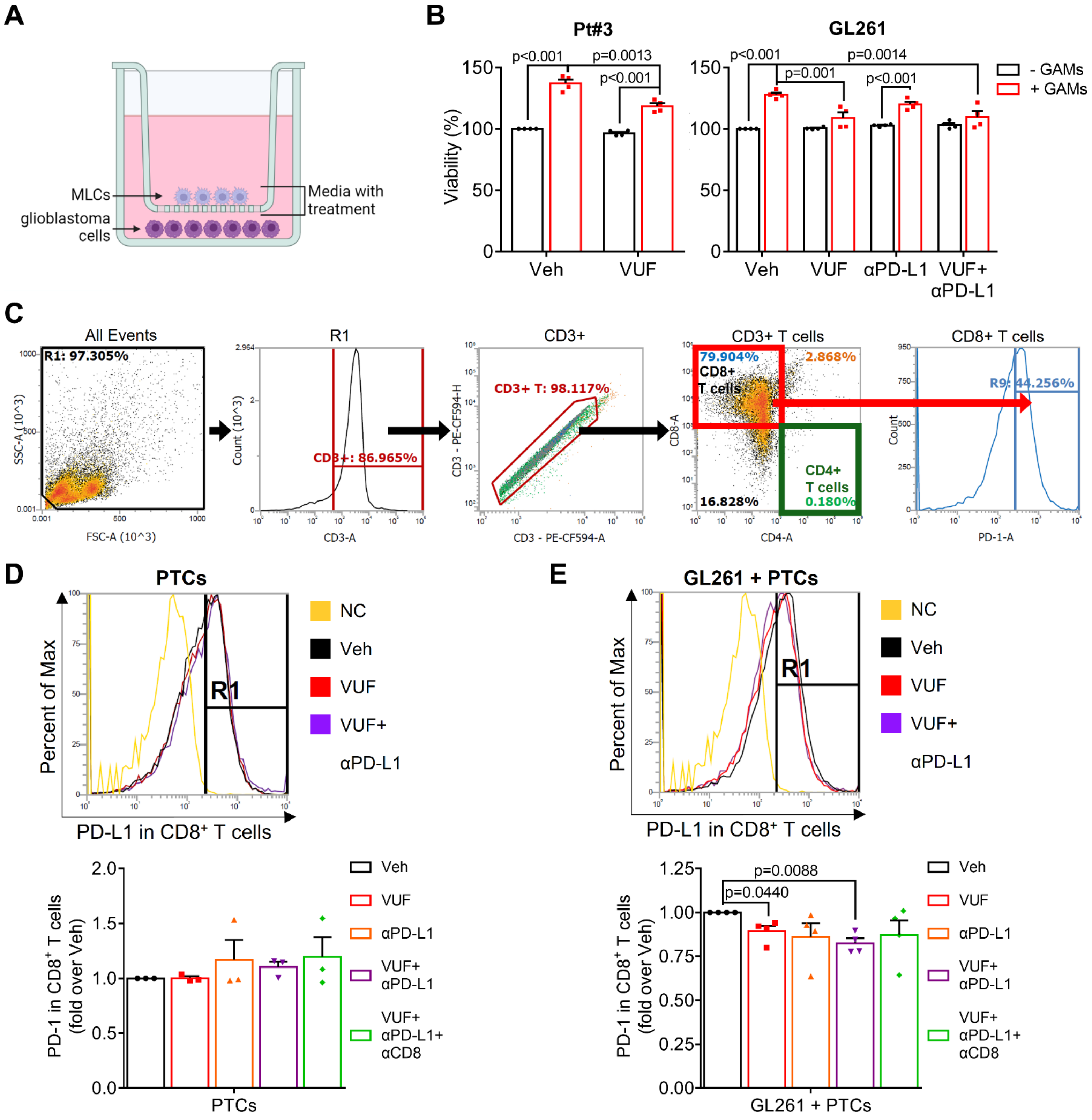

Figure S7. The effects of VUF-αPD-L1 combination on tumor size and body weight

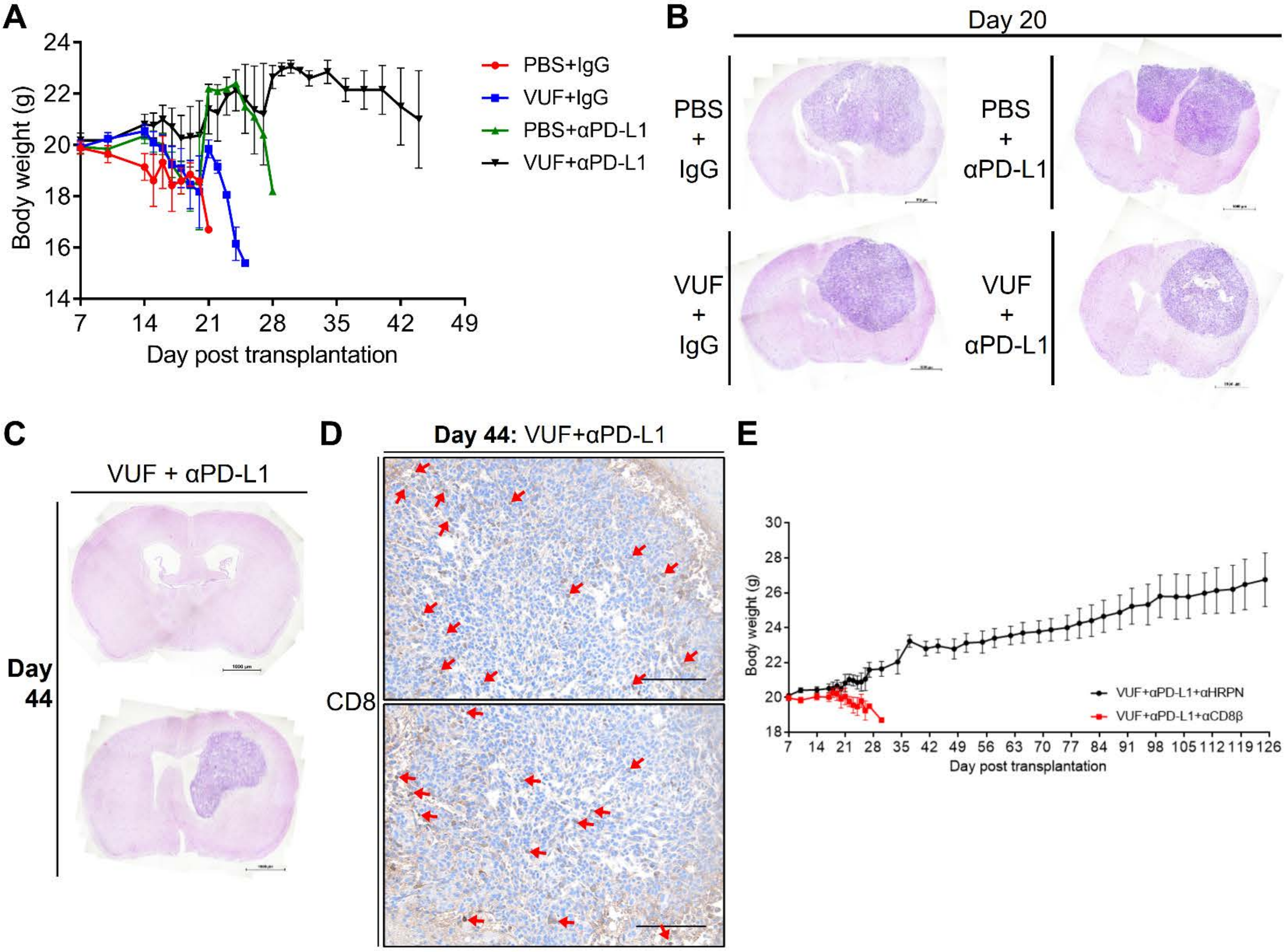

Supplement: Supplementary file 4 — Supplementary Figure S1-S7 [file 41419_2024_6784_MOESM4_ESM.pdf]
