# Supplementary material for: CXCR7 activation evokes the anti-PD-L1 antibody against glioblastoma by remodeling CXCL12-mediated immunity
Source: Cell Death Dis. 2024 Jun 19;15(6):434. doi: 10.1038/s41419-024-06784-6 (PMC11187218; doi:10.1038/s41419-024-06784-6)

Original blots for Figure 3F

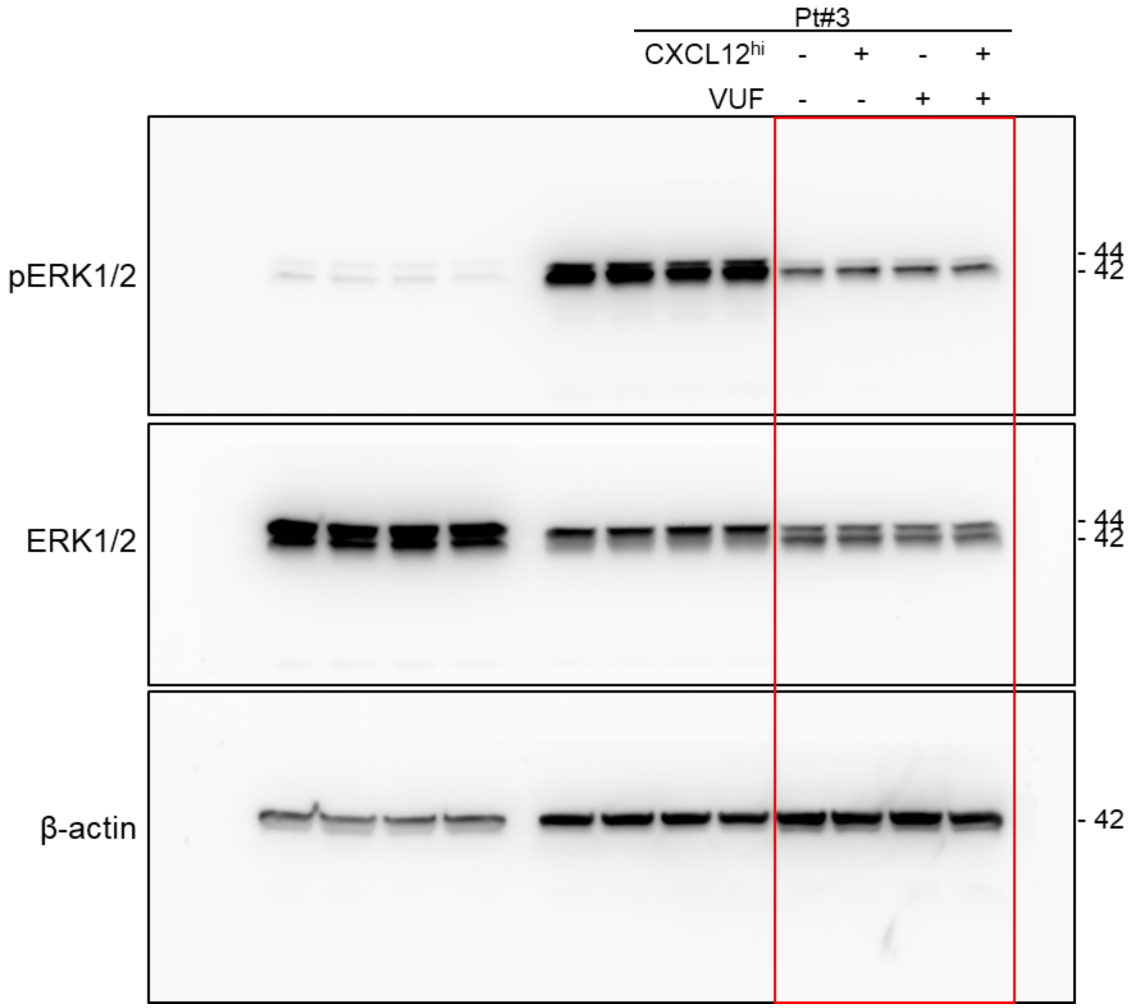

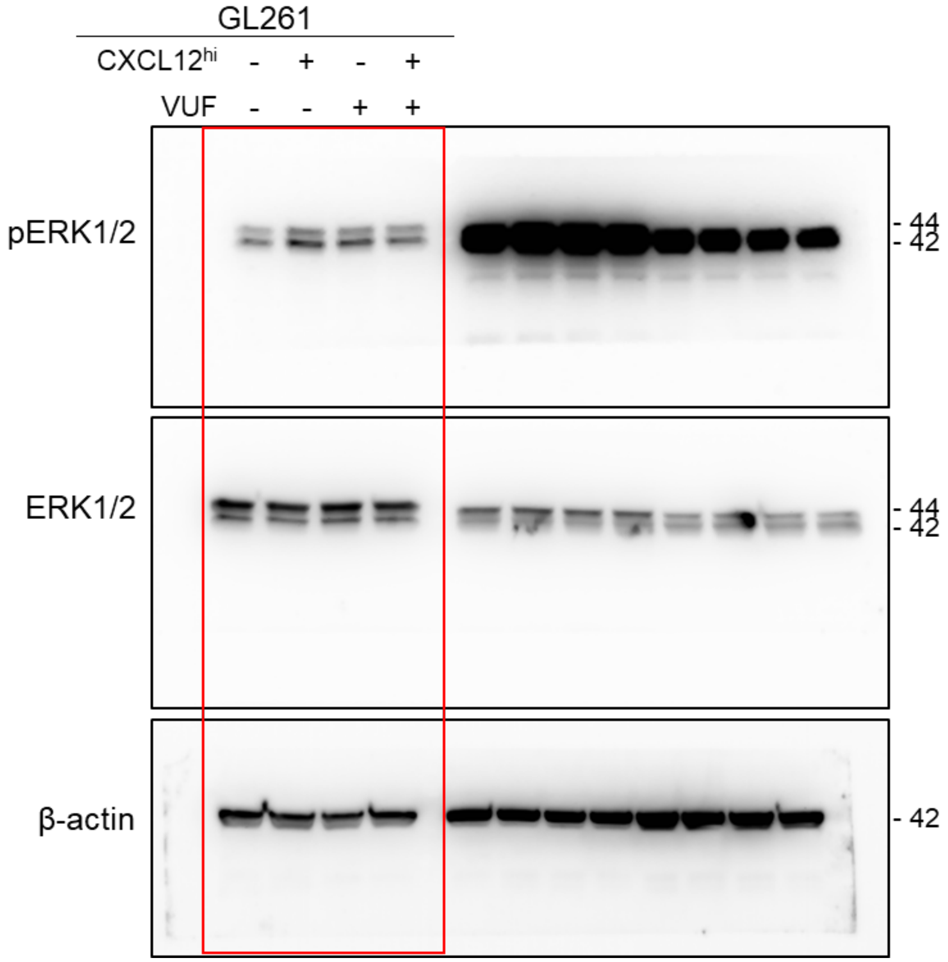

Original blots for Figure S2E

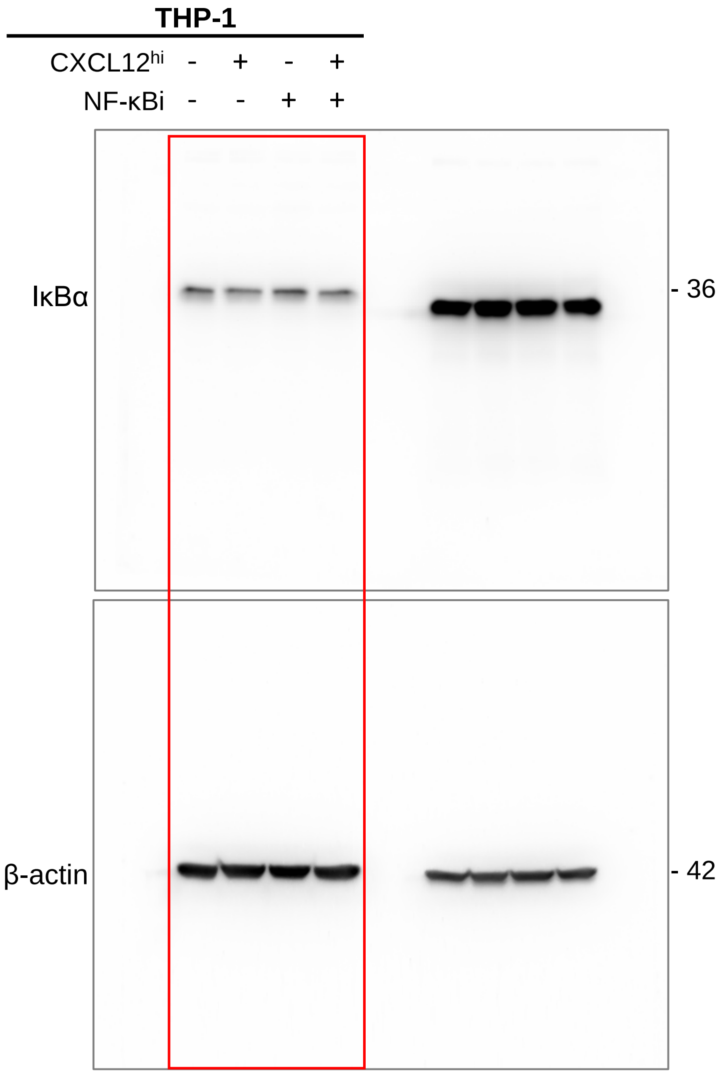

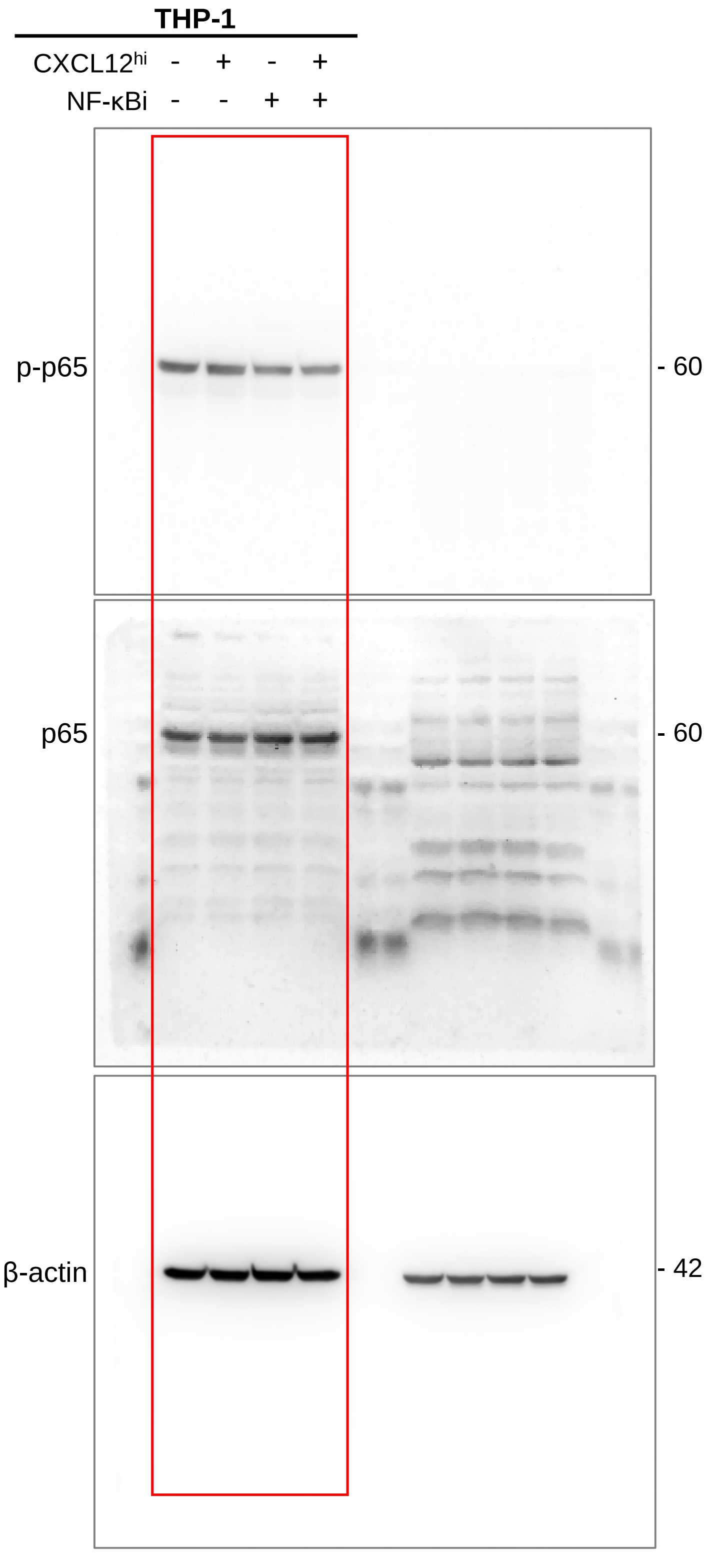

Original blots for Figure S3B

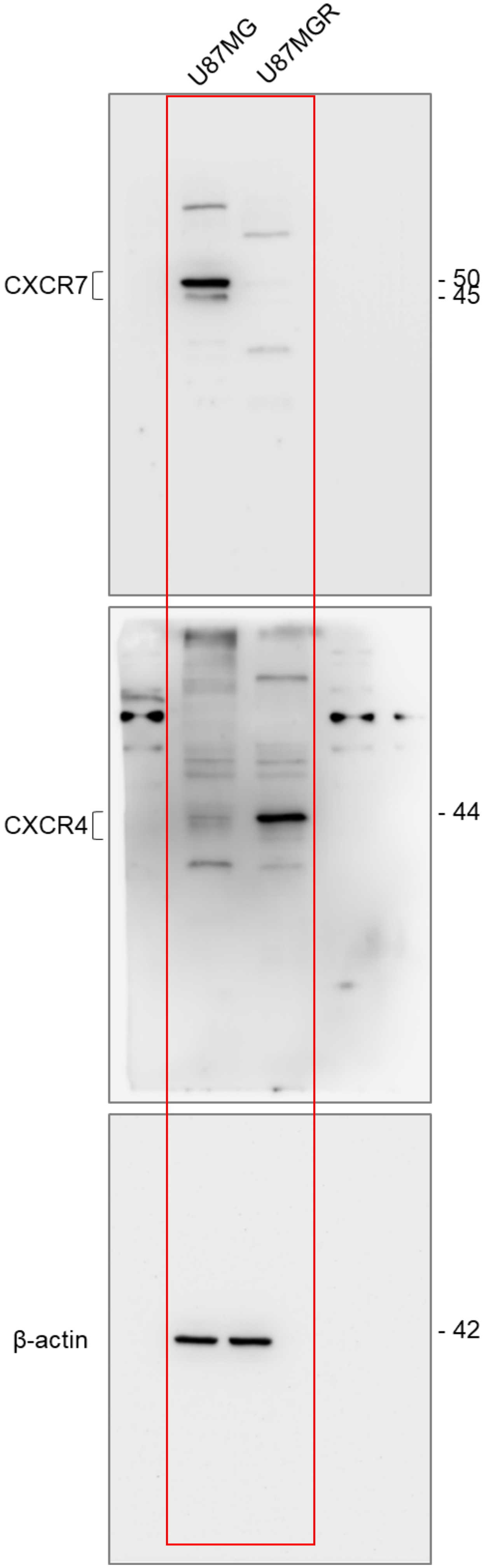

Original blots for Figure S3H

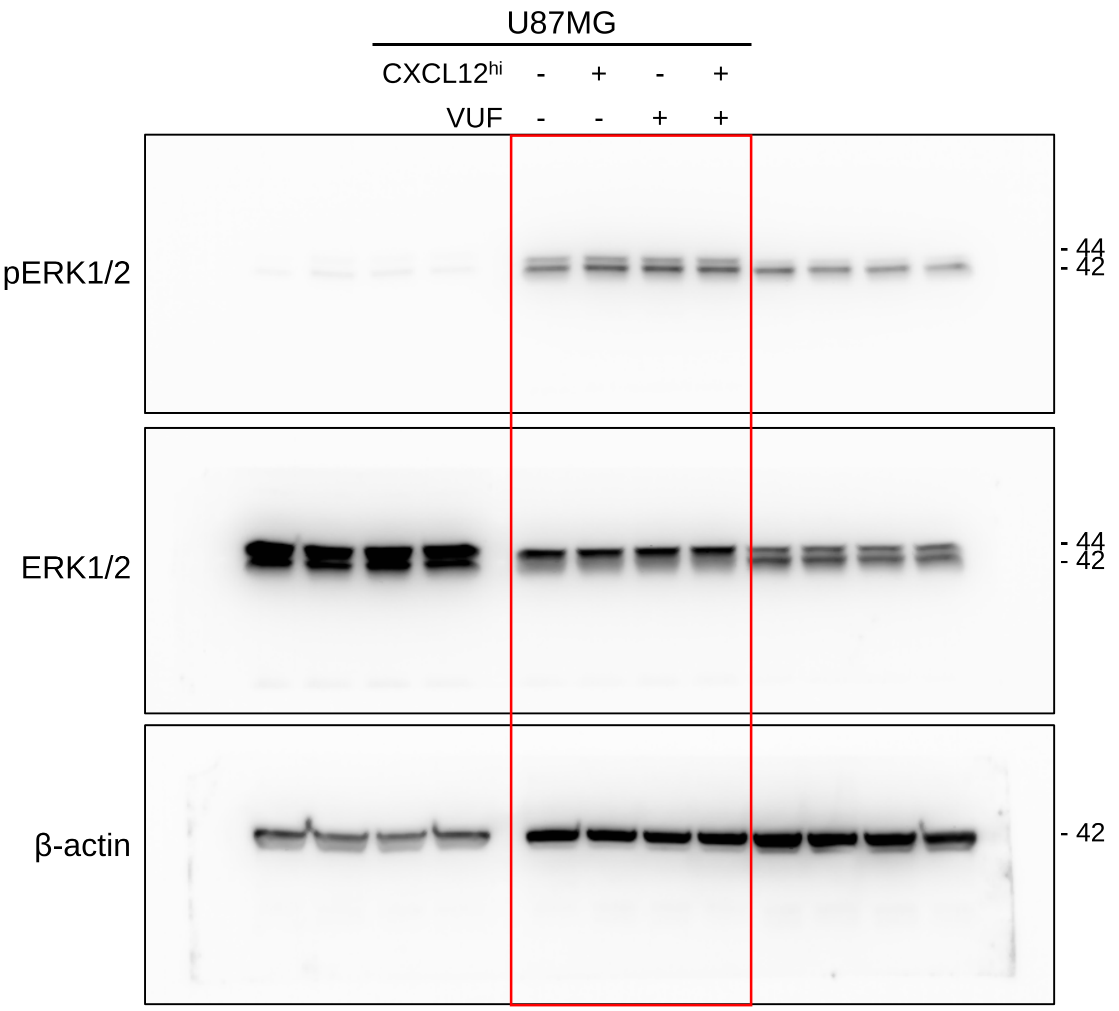

Supplement: Supplementary file 5 — Original data file [file 41419_2024_6784_MOESM5_ESM.pdf]
